# Shielded magnetic small-angle neutron scattering for characterization of radioactive samples

**DOI:** 10.1107/S1600576725003176

**Published:** 2025-05-31

**Authors:** Kevin G. Field, Caleb P. Massey, Kurt R. Smith, Samuel A. Briggs, Dalong Zhang, Kenneth C. Littrell

**Affiliations:** a Nuclear Engineering and Radiological Sciences University of Michigan–Ann Arbor 2301 Bonisteel Boulevard Ann Arbor MI48109 USA; b Oak Ridge National Laboratory Oak Ridge TN37831 USA; c Y-12 National Security Complex, Oak Ridge, TN37830, USA; d Oregon State University Corvallis OR97331 USA; e Baylor University Waco TX76706 USA; HPSTAR and Harbin Institute of Technology, People’s Republic of China

**Keywords:** FeCrAl, small-angle neutron scattering, SANS, magnetic scattering, radioactive materials characterization

## Abstract

The development of a Pb-shielded fixture for the execution of a small-angle neutron scattering (SANS)-based workflow for interrogation of highly irradiated nuclear materials has been explored. The Pb shielding was specially designed to reduce the detected radioactivity from the specimen during SANS experiments, and the overall configuration is termed shielded magnetic SANS (SM-SANS). Two FeCrAl-based alloys, C35M and 125YF, were examined with the SM-SANS technique using a free-form size distribution locally monodisperse model in both the as-received and irradiated states. Quantitative values derived from the free-form size distribution were compared with atom probe tomography experiments. Microstructural and compositional parameters determined using the two characterization techniques were complements of each other. The results demonstrate that the SM-SANS technique is an effective means of characterizing nanoscale clustering in irradiated material systems and provides new avenues for investigating radioactive material microstructures.

## Abstract 2

The study presents a novel shielded magnetic small-angle neutron scattering (SM-SANS) technique using lead shielding to facilitate the nanoscale characterization of clustering and precipitation in highly radioactive nuclear materials. By comparing the results with atom probe tomography, the research demonstrates that SM-SANS effectively quantifies microstructural parameters and provides compositional insights, thereby offering a viable and safe method for analyzing irradiated nuclear alloys.

## 1. Introduction

Characterization of nanoscale clustering and precipitation is a common task within generalized materials science. A range of tool sets exist for the quantitive characterization of the size, number density and volume fraction of these microstructural features, including transmission electron microscopy (TEM), atom probe tomography (APT) and small-angle scattering using either X-rays (small-angle X-ray scattering, SAXS) or neutrons (small-angle neutron scattering, SANS). For most characterization efforts, the limiting factors for using these tool sets in an experiment are the ability to perform adequate sample preparation and/or access to the instrument(s). In the sub-class of radioactive nuclear materials, access to instruments is often the greater issue. Under neutron irradiation, and sometimes under other forms of energetic irradiation, samples form unstable activation products, leading to the emission of hazardous radiation from the sample(s). The type and intensity of this hazardous radiation is dependent on a wide range of factors, including but not limited to sample composition, energetic source energy spectrum and sample volume. Successful studies of nuclear materials ultimately require neutron or energetic bombardment to study the nanoscale clustering and precipitation changes in the material for common industrial deployments, and hence such deleterious sample activation cannot be eliminated.

If the harmful radiation in nuclear materials exceeds a given background and/or release value(s), then the sample is considered radioactive, or *hot* in vernacular terms. These conditions commonly preclude the use of open-access TEM, APT and scattering equipment because of safety and contamination concerns. This exclusion means that special­ized/dedicated machines and/or facilities are necessary to perform nanoscale quantification of activated nuclear mater­ials. These requirements increase experimental costs and limit access to state-of-the-art quantification techniques. Since the early 2000s, the inclusion of focused ion beam (FIB) sample preparation in the workflow of these radiological facilities has enabled production of high-quality specimens with a significantly reduced sample volume for APT and TEM. This volume reduction typically results in sample radiation levels below the given background and/or release values, and this sample reduction and associated radiation reduction strategy have ultimately opened up new pathways for cutting-edge nano­scale characterization of irradiated nuclear materials. However, both APT and TEM have intrinsic limitations, such as limited sample volume, ambiguity in quantification from various artifacts [*e.g.* aberration issues in APT (Gault *et al.*, 2012 ▸; Hyde *et al.*, 2014 ▸; Hatzoglou *et al.*, 2019 ▸)] and so on.

The limitations in TEM and APT indicate that greater insight into nanoscale clustering and precipitation is gained when they are used in parallel with another complementary technique such as SAXS or SANS. Many nuclear materials studies have highlighted the advantage of such an approach (Hyde *et al.*, 2014 ▸; Briggs *et al.*, 2017 ▸; Simm *et al.*, 2017 ▸; Cunningham *et al.*, 2014 ▸). This type of idealized approach is not usually feasible for highly radioactive specimens. For example, a dose rate of 100 mrem h^−1^ at 30 cm from the sample’s surface is a common designation limit in US facilities. Scattering techniques require volumes that are significantly larger (>10^−7^ m^3^) than common FIB sample volumes (∼10^−17^ m^3^) for TEM and APT. Therefore, this sample volume reduction strategy, which lowers specimen radioactivity for TEM and APT, is not typically feasible for SAXS, SANS and other bulk analysis techniques.

One method yet to be widely explored for reducing the radioactive hazard of SANS or SAXS investigations on activated nuclear material samples is the use of shielding. Shielding is frequently used in sample transportation, storage and handling, and shielding materials commonly have high atomic numbers (*e.g.* Pb or W). These high-*Z* materials can effectively attenuate the energetic radiation emitted by the sample. Note that such materials can promote additional radiation via the production of *Bremsstrahlung* radiation, but the overall radiation escaping the shielding is generally still lower than in unshielded specimens. SANS provides a unique opportunity to use Pb-based shielding because the neutron incoherent scattering cross section is low (0.003 barns) but still effectively shields common α-, β- and γ-ray energies of activated nuclear materials. The theoretical result is that a sample can be shielded, yet transparency is still provided for the analyzing neutron beam in SANS. This approach would enable complementary characterization of highly radioactive specimens.

This work details the first iteration of techniques developed for using Pb-shielded SANS for nanoscale characterization of clustering and precipitation in radioactive nuclear material specimens. The shielding containers were designed specifically to reduce the radiation of the specimen-holder configuration while facilitating loading in a radiological handling facility. The individual specimen shielding containers were also designed to enable use of a saturating magnetic field to separate the magnetic and nuclear scattering cross sections, hence the designation shielded magnetic SANS, or SM-SANS for short. Analysis of the SM-SANS technique is completed using three separate models to evaluate both the method and the analysis technique. These three models are the monodisperse approximation, the log-normal size distribution form and a free-form size distribution within the assumed local monodisperse approximation model for spherical precipitates (Pedersen, 1994 ▸, 1997 ▸). The results of the SM-SANS analysis are compared directly with APT results previously provided on the samples of interest, to enable an assessment of the effectiveness of the SM-SANS technique.

## 2. Experimental

### 2.1. Specimens and irradiation experiment

The materials that were neutron irradiated and used in subsequent small-angle scattering experiments (Table 1 ▸) represent two variants of the iron–chromium–aluminium (FeCrAl) alloy class. The first, C35M, is a wrought FeCrAl alloy that does not exhibit nanoscale precipitation or alloy clustering in the as-received state (Gussev *et al.*, 2017*a* ▸; Yamamoto *et al.*, 2015 ▸). The second, 125YF, is a powder-metallurgy-derived FeCrAl alloy that is processed in a manner that forms a high density (>10^23^ m^−3^) of small Y–Al–O nano-clusters (<10 nm) throughout the matrix (Dryepondt *et al.*, 2018 ▸; Massey *et al.*, 2019*b* ▸). Under neutron irradiation at a relevant temperature (*e.g.* near 330°C), both alloys are known to exhibit enhanced precipitation of the Cr-rich α′ phase as a result of a miscibility gap in the Fe–Cr–Al phase diagram (Massey *et al.*, 2019*b* ▸; Kobayashi & Takasugi, 2010 ▸; Zhang *et al.*, 2019 ▸). In the case of the 125YF specimen, previous APT results have shown that the nano-oxides remain within the matrix after irradiation under conditions identical to those used in this study (Massey *et al.*, 2019*b* ▸).

The size and number density of the Cr-rich α′ and Y–Al–O clusters are known to impact the radiation tolerance of these alloys, resulting in hardening (*e.g.* yield strength increase) of the material after irradiation (Field *et al.*, 2015 ▸). The clusters typically reside within the 0–15 nm scale range (Briggs *et al.*, 2017 ▸; Massey *et al.*, 2019*b* ▸; Field *et al.*, 2015 ▸; Dryepondt *et al.*, 2014 ▸), are spherical in nature and are also assumed to be antiferromagnetic in the ferromagnetic matrix of the FeCrAl alloy. Both nanoscale precipitates provide ideal features to probe using the SM-SANS technique because of their size and morphologies, while having direct relevance to their performance under irradiation. Therefore, these two materials were selected for neutron irradiation and small-angle scattering experimentation.

The processing conditions and general microstructure of the C35M feedstock are identical to those reported in previous studies (Gussev *et al.*, 2017*a* ▸, 2018 ▸; Mao *et al.*, 2022 ▸) as well as for the 125YF alloy (Dryepondt *et al.*, 2018 ▸; Massey *et al.*, 2019*b* ▸). Feedstocks of the C35M and 125YF alloys were used to machine dog-bone sheet-type SS-J2 specimens (nominal tensile head size of 4.0 × 4.1 × 0.5 mm) (Gussev *et al.*, 2017*b* ▸). Specimens were neutron irradiated to a total nominal damage dose of 1.8 displacements per atom (dpa) with an average irradiation temperature estimated at 357°C based on dilatometric analysis of SiC specimens (Field *et al.*, 2019 ▸) co-irradiated with the FeCrAl tensile specimens. Irradiations were carried out in the High Flux Isotope Reactor (HFIR) at Oak Ridge National Laboratory (ORNL) in the flux trap positions for a nominal dose rate of 9.3 × 10^−7^ dpa s^−1^. Further details regarding the irradiation and test configurations are reported in previous studies of the same samples (Massey *et al.*, 2019*b* ▸, 2021 ▸; Gussev *et al.*, 2018 ▸).

Post-irradiation, the dog-bone sheet-type specimens were transferred to a remote-handling radiologically shielded hot cell facility equipped with an Instron universal testing machine. Tensile testing was performed at room temperature at a nominal strain rate of 10^−3^ s^−1^ using a shoulder-loaded configuration which is known to induce insignificant strain within the tensile head of common metallic alloys up to and at fracture (Gussev *et al.*, 2017*b* ▸). After tensile testing, the specimens were transferred to a high-radiation direct-handling area for radiological characterization and Pb encapsulation prior to shipment and analysis on HFIR’s General-Purpose SANS (GP-SANS) beamline (Wignall *et al.*, 2012 ▸). No sample preparation (*e.g.* metallographic grinding/polishing) was per­formed between irradiation and Pb shielding encapsulation.

### 2.2. SANS configurations

**Figure 1:**
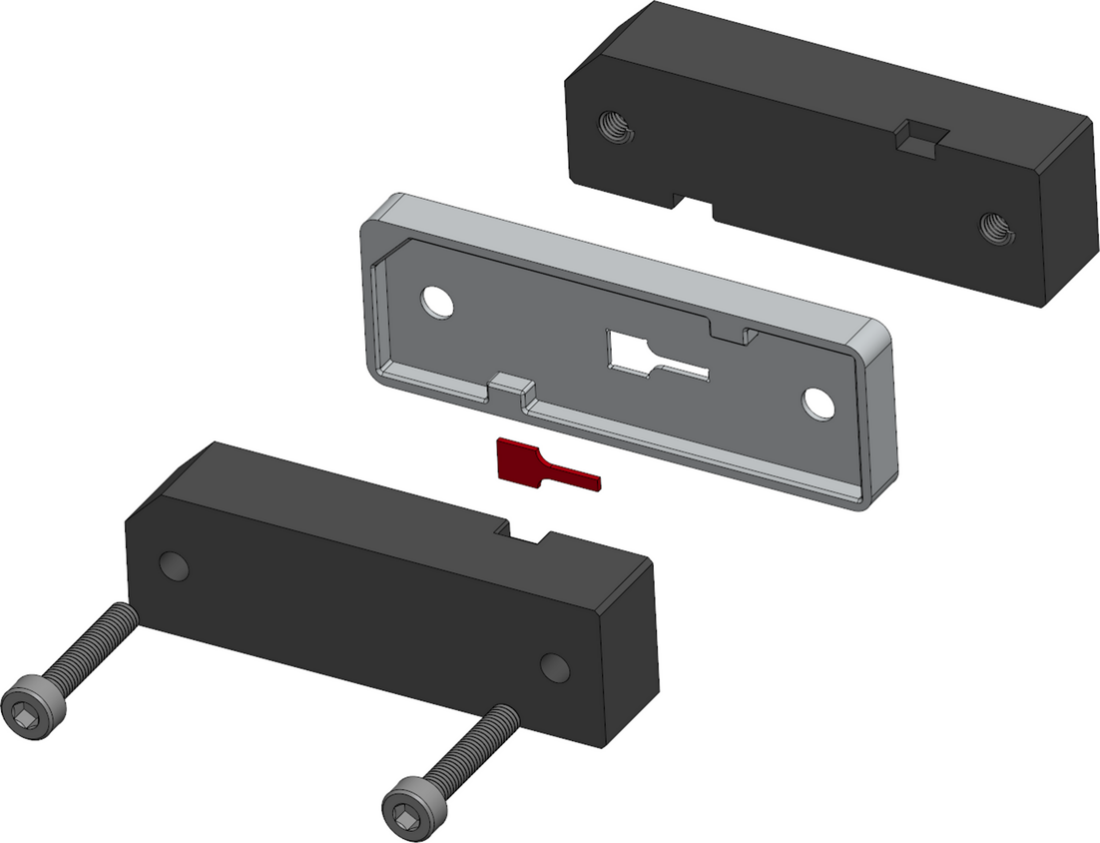
Exploded view of the Pb-shielded specimen carrier for highly radioactive specimens for SM-SANS analysis. Tensile half specimen highlighted in red and Pb-based shielding highlighted in dark gray.

The Pb shielding was designed to accommodate half of the broken tensile specimens using a stainless steel blank machined to an oversized half tensile dimension. The blank was keyed to allow sandwiching within Pb shields in only a single orientation. The configuration of the stainless steel blank and Pb shields is shown in Fig. 1 ▸. The Pb shields were cast and then subsequently machined to the configurations shown in Fig. 1 ▸ from high-purity Pb feedstock. Final encapsulation included applying household electrical tape around the sandwiched portion of the stainless steel blank to reduce radioactive contamination concerns during beamline handling. Measurements of the γ-irradiation dose before and after Pb shielding saw a reduction of roughly 20–25% in the detected activity at 30 cm from the sample’s surface. The highest dose rate was measured on the side where only the stainless steel blank was shielding the specimen. Future redesigns of the shielding configuration could eliminate this effect and further reduce the externally detected activity of highly radioactive specimen-holder configurations.

A specialized holder was designed and retrofitted to the CG-2 GP-SANS beamline at HFIR (Wignall *et al.*, 2012 ▸) to enable effective loading and unloading of the shielded specimens. Fiducial marks were made on the Pb shielding to facilitate sample alignment because the sample orientation cannot be determined after encapsulation. Shielded samples were manually loaded individually on the beamline. Both irradiated and unirradiated reference samples were placed within the shielded holders, while an additional shielded configuration with no sample was also used. The shielded holders were placed in a saturating magnetic field (*H*) of ∼2 T perpendicular to the incident neutron beam to separate the magnetic and nuclear scattering cross sections. All measurements were made at ambient temperature.

The beamline for the 125YF specimens was configured for three different detector distances: two using 0.8 nm neutrons at distances of 1.5 and 9.0 m, and a third configuration using 1.2 nm neutrons at a distance of 19.3 m. The beamline for the C35M specimens was configured using 0.6 nm neutrons at distances of 1.2 and 8.0 m, with an additional configuration using 1.2 nm neutrons at a distance of 19.3 m. The configurations were selected to minimize Bragg diffraction streaks or flares on the detectors; although the Pb used was of high purity, Bragg diffraction was still observed from the Pb shielding. The configurations allowed for continuous data spanning *q* ranges of 0.0000658 ≤ *q* ≤ 0.0248 nm^−1^ for the 125YF specimens and 0.00572 ≤ *q* ≤ 0.0433 nm^−1^ for the C35M specimens, where *q* is defined as *q* = (4π/λ) sin θ, θ is half the scattering angle and λ is the wavelength of the incident radiation.

### 2.3. Application of the local monodisperse approximation to SM-SANS data

In this work, the local monodisperse approximation (LMA) proposed by Pedersen (1994 ▸, 1997 ▸) was applied to the 1D data sets, in which the small-angle scattering intensity is given by
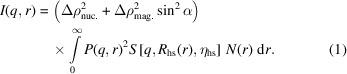
Here, Δρ_nuc., mag._ is the nuclear (nuc.) or magnetic (mag.) contrast between the clusters and the matrix, α is the angle between the magnetization of the sample and the scattering vector **q**, *P*(*q*, *r*) is the particle form factor for spherical scatterers (Pedersen, 1994 ▸, 1997 ▸), *S*(*q*, *R*_hs_, η) is the structure factor of the monodisperse hard-sphere model (Kinning & Thomas, 1984 ▸), and *N*(*r*) is the number-density size distribution with radius *r*. For brevity, we refer the reader to the literature for the written forms of the spherical form factor (Pedersen, 1997 ▸) and the monodisperse hard-sphere structure factor (Kinning & Thomas, 1984 ▸). *R*_hs_ is the hard-sphere radius and η_hs_ is the volume fraction of hard spheres. The hard-sphere radius can be directly related to the scattering cluster particle radius *r* by a constant *C*_hs_, where
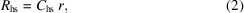
whereas the volume fraction of hard spheres can be related to the scattering cluster volume fraction by
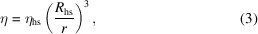
as long as the sample exhibits a locally monodisperse behavior.

The LMA approach simplifies the SANS analysis by assuming that, within a local volume, the scattering clusters are surrounded by clusters of identical size and the variation from one local volume to another changes slowly. Thus, the overarching assumption is that the position and size of the clusters within the matrix are fully correlated. The even more simplified monodisperse approximation (*e.g.* non-localized or globalized monodispersion) has been applied previously to the FeCrAl system and shown to give a reasonable match to trends obtained with correlated APT studies (Briggs *et al.*, 2017 ▸; Field *et al.*, 2015 ▸, 2018 ▸), thus providing a justification for expanding into the more detailed LMA approach here.

The clusters in the C35M and 125YF alloys were initially considered to be antiferromagnetic clusters in a ferromagnetic matrix. The ferromagnetic nature of the matrices enables the samples to be analyzed with SANS using two contributions to the SANS intensity – nuclear and magnetic – when placed in a saturating magnetic field. The nuclear or magnetic scattering contrast takes the form (Mathon *et al.*, 2012 ▸)
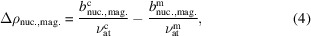
where 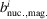 (*i* = m, c) is the mean nuclear or magnetic scattering length in the clusters (c) or in the matrix (m) and 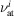 is the mean atomic volume. The mean magnetic scattering length of the clusters 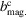 was initially assumed to be equal to zero in the model development and analysis, but as will be demonstrated in the discussion below, a modification of the mean magnetic scattering length of the clusters could be considered for analyzed systems. The mean magnetic scattering length of the Fe-based matrix 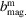 is written in a simplified form (Bacon, 1975 ▸) as
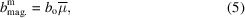
where *b*_o_ is a constant with a value of 2.7 × 10^−13^ cm and 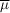 is the mean magnetic moment of the atoms in Bohr magneton units (μ_B_). The result is that the magnetic contrast between the clusters and the matrix is directly correlated to two parameters, 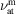 and 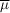, both of which have been shown to be directly related to the FeCrAl matrix composition, particularly the Cr and Al contents (Blau *et al.*, 1977 ▸; Chang *et al.*, 2019 ▸; McMurray *et al.*, 2017 ▸).

**Figure 2:**
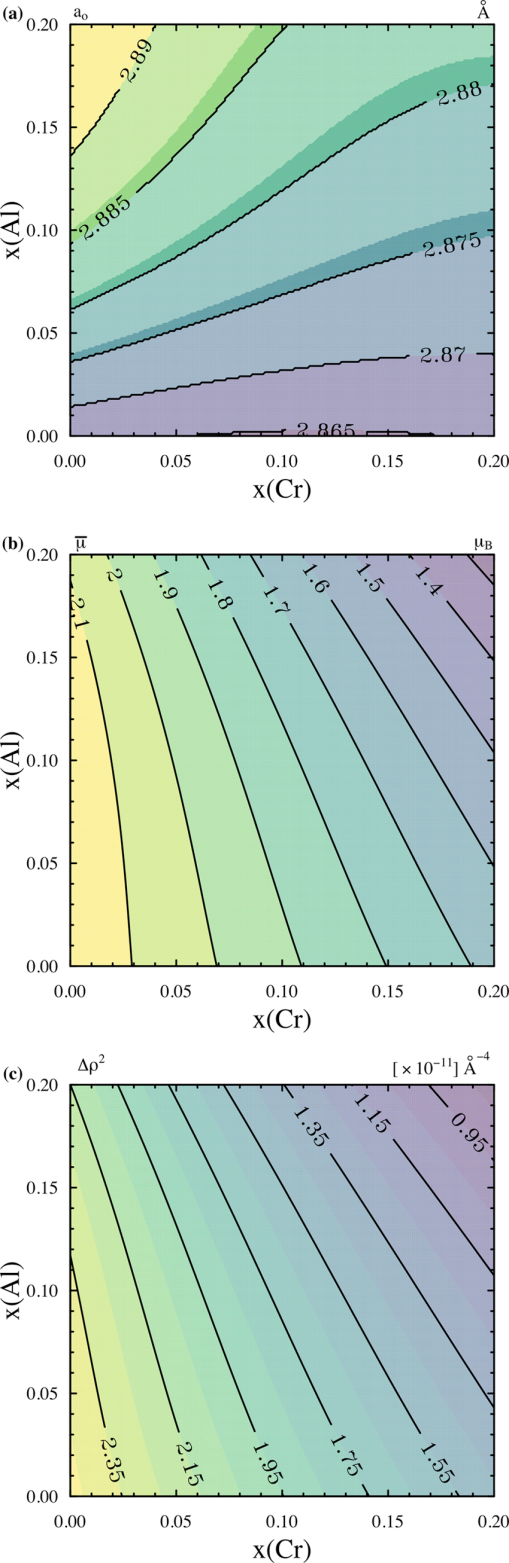
Calculated properties of the α-FeCrAl phase used within the FF and LN local monodisperse approximation fitting model.

The calculation of Δρ_mag._ is necessary to determine quantitively the number density and volume fraction of the clusters using equation (1) when the scattering cross-section data are presented on an absolute scale. Therefore, the compositional dependence on 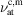 and 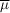 must be considered. The composition dependence of 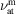 is related to the lattice parameter *a*_0_, because 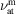 can be calculated as
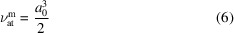
when the body-centered cubic (b.c.c.) structure of the FeCrAl matrix is taken into account. The lattice parameter variation was assumed to depend only on the Fe, Cr and Al content of the C35M and 125YF alloys; minor alloy element contributions were not considered. The determination of the lattice parameter as a function of Cr and Al content was approximated using a Redlich–Kister series (Redlich & Kister, 1944 ▸),
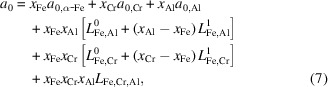
where *x*_Fe_, *x*_Cr_ and *x*_Al_ are the model fractions of Fe, Cr and Al in the matrix, respectively, *a*_0,α-Fe_, *a*_0,Cr_ and *a*_0,Al_ are the lattice parameters of pure α-Fe, Cr and Al, respectively, and *L*_*i*_ (*i*, *j*, *k* = Fe, Cr, Al) are binary (*i*, *j*) and ternary interaction parameters (*i*, *j*, *k*). The interaction parameters were fitted to 62 experimentally determined values spanning an Fe mole fraction of 0.5 to 1, a Cr mole fraction of 0 to 0.3 and an Al mole fraction of 0 to 0.3 (Preston, 1932 ▸; Taylor & Jones, 1958 ▸; Abrahamson & Lopata, 1966 ▸). The fitted interaction parameters are provided in the supporting information along with the lattice parameters (Table S1). The resulting change in the lattice parameter of the FeCrAl matrix as a function of Cr and Al content is plotted in Fig. 2 ▸(*a*).

The composition dependence of the mean magnetic moment 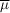 for FeCrAl alloys was empirically determined by Blau *et al.* (1977 ▸) and is adopted here. The empirical fit can be written as (Blau *et al.*, 1977 ▸)
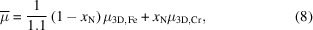
and
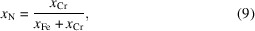

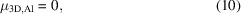

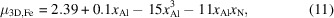

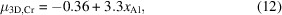
where μ_3D,*i*_ (*i* = Fe, Cr, Al) represents the localized atomic moments. The empirical fitting completed by Blau *et al.* (1977 ▸) estimates the error of the fit to ±0.06 μ_B_. The resulting change in the mean magnetic moment as a function of the Cr and Al contents in the FeCrAl matrix is plotted in Fig. 2 ▸(*b*).

By substituting the composition-dependent parameter equations [equations (6)–(12)] into equation (5) and subsequently into equation (4), the magnetic scattering contrast for any Fe-rich FeCrAl system can be estimated. This estimation for the Fe-rich corner of the FeCrAl phase diagram is plotted in Fig. 2 ▸(*c*), with the values used in the fitting of equation (1) provided in Table 2 ▸ for the C35M and 125YF alloys assuming a normalized Fe, Cr, Al matrix with no minor alloying additions. Although the bulk Cr and Al contents only vary by several atomic percent (Table 1 ▸), the lattice parameter and mean magnetic moment vary enough to alter the magnetic scattering contrast and thus the final quantification of the number density and volume fraction of the clusters between the two alloys.

The size distribution in equation (1) was determined using a three-stage approach. The first stage is the approximation of the radius of the clusters present in the material via the non-localized monodisperse approximation used previously for FeCrAl alloys. This approximation is designated herein as the MD approach (Pedersen, 1994 ▸), and it only provides an approximation of the mean size, number density and volume fraction of scattering clusters in the matrix; no details on the size distribution are available. The second stage approximates the full size distribution using the log-normal distribution, an approach analogous to that used by Massey *et al.* (2019*a* ▸) on similar alloys. This stage/approach, designated here as the LN approach, restricts the determination of the size distribution to a single log-normal distribution. Bimodal or more complex distributions are not considered. In general, this distribution has proven effective in approximating cluster morphologies in FeCr and FeCrAl alloys (Massey *et al.*, 2019*a* ▸; Ohnuma *et al.*, 2009 ▸; Han *et al.*, 2014 ▸), but additional nuances in the size distribution which arise from irradiation cannot be effectively captured because of the restrictive nature of the presumed size distribution shape.

To overcome the limitations of the MD and LN approaches, the free-form or FF approach used by Pedersen (1994 ▸) and Glatter (1977 ▸, 1980 ▸) was added as a third stage using the inputs from the MD and LN stages. The FF approach constrains the size distribution to include only positive values and a smooth shape, but the form of said shape in the size distribution is not constrained. To accomplish this, the FF approach approximates the size distribution as a linear combination of a set of basis functions. The number density size distribution of the FF approach can then be written as
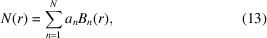
where *a*_*n*_ are the coefficients and *B*_*n*_ are the basis functions; here, the basis functions are a series of normalized Gaussians. A detailed explanation of the background and application of the linear combination of a set of basis functions can be found in the literature (Pedersen, 1994 ▸; Hansen & Pedersen, 1991 ▸). The same methods were implemented in this study. The size distribution in equation (13) is defined from zero to *R*_max_. The coefficients *a*_*n*_ are determined using a least-squares routine that includes a regularization parameter λ to constrain the smoothness of the size distribution (Glatter, 1980 ▸) and a non-negativity constraint. The regularization parameter can be determined using the point inflection method proposed by Glatter (1977 ▸). Details of the computer fitting routine for the least-squares routine to the SM-SANS data are provided in the following section.

The FF approach requires user-defined values for *R*_max_, which is the upper limit of the size distribution function, and either the resolution in real space Δ*R* or the number of cubic spline functions in equation (13), *N* (or *N*_SANS_; see Section 2.5). The three parameters, *R*_max_, Δ*R* and *N*, are interrelated by
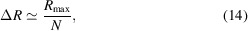
and are then related to the sample *q* range by
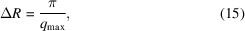
where 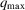 is the maximum *q* in which the LMA approximation is being applied to the 1D data. In this work, Δ*R* is not fixed and is based strictly on equation (15). To eliminate the need for a user-defined value for *R*_max_, the value is determined by extracting details from the MD stage and is simply computed by assuming that the total size distribution is four times the radius of scatterers evaluated in the MD stage rounded to the nearest tenth of a nanometre. With *R*_max_ and Δ*R* defined from the MD stage and from the experimental configurations, respectively, *N* was found via equation (14). It was found empirically that the general fit of the FF stage was improved with a slightly increased number of splines, so a standard modifier of adding three more splines was used, or effectively
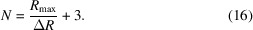


In the LN and FF approaches, the values for the mean radius of clusters, volume fraction and number density can be determined using the moments 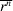 of the size distribution *N*(*r*), where
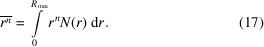
The number density of the scattering clusters is defined as
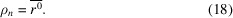
The mean radius of the scattering clusters is
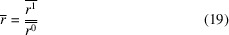
and the volume fraction is
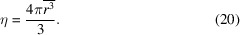
Additional information can be determined from the moments of the size distribution, including the radius of gyration, surface area, skewness of the distribution and kurtosis; the equations related to these are provided in the literature (Sequeira *et al.*, 1995 ▸).

The application of the saturating external magnetic field allows for separation of the nuclear and magnetic scattering components [equation (1)]. This separation provides the ability to interrogate the composition of the scattering clusters on the basis of the ratio between the nuclear and magnetic scattering contrast. This ratio, commonly referred to as the *A* ratio, can be written as (Mathon *et al.*, 2003 ▸)
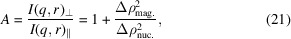
where 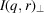 is the intensity perpendicular to the saturating magnetic field and 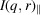 is the intensity parallel to the saturating magnetic field. For both scattering contrasts the values are composition dependent, so the *A* ratio provides an indirect means of assessing the composition of the scattering clusters and the surrounding matrix. The values of 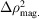
are calculated via the method described previously, and 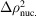 is empirically fitted as described in the following section, to extract the volume-averaged *A* ratio of scatterers in a given specimen.

### 2.4. Computer-fitting routine to SM-SANS data

The complexity in the LMA approximation when applied to both the nuclear and magnetic scattering data required the development of an automated computer-fitting routine to extract quantitative information regarding the clusters in the FeCrAl matrix, including the size, number density, volume fraction and composition of the clusters. The routine was built using the R programming language on build R 4.2.2 GUI 1.79 Big Sur ARM (8160) with parallelized routines. The magnetic scattering was estimated by subtracting the combined magnetic and nuclear scattering [*i.e.* subtracting 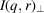 from the nuclear scattering 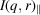]. The routine used the estimated magnetic scattering in text (*.txt) or comma-separated format (*.csv) and performed the primary three-stage fitting approach. The combined magnetic and nuclear scattering, as well as the nuclear scattering, are also input into the routine with an *I*(*q*) ∝ *Aq*^−*m*^ + *B* inverse power law background subtracted from both data sets. In the *I*(*q*) ∝ *Aq*^−*m*^ + *B* background, *A* is the power law length scale factor, *m* is the power law exponent (typically near *m* = 4) and *B* is the measured background intensity.

The routine uses the three-stage approach to find first the fitted non-localized monodisperse approximation and log-normal size distribution, and then conducts the FF fitting based on equation (1). The approach is different from that of Pedersen but similar in design to that used by Tsao *et al.* (1999 ▸). The overall fitting and output process includes seven primary steps, as presented below.

*Step 1.* Perform the MD fitting stage on the magnetic scattering cross-section data to derive *R*_max_ and starting values for the log-normal size distribution, as well as *C*_hs_ and η_hs_. This step is performed first because of the simplicity of the MD fitting procedure, with limited fitting failures throughout the routine.

*Step 2.* Perform the LN fitting stage on the magnetic scattering cross-section data to establish refined starting values for *C*_hs_ and η_hs_ in the FF approach and to determine the mean and standard deviation of the log-normal distribution for comparison with the FF approach.

*Step 3*. On the basis of the determined values in Steps 1 and 2, estimate the stabilizing parameter λ using the point inflection method (Glatter, 1977 ▸) and the FF approach on the magnetic scattering cross-section data.

*Step 4.* Perform a parallelized grid search using the FF method to determine the optimal values of *C*_hs_ and η_hs_ while λ is held constant, where λ is determined from Step 3.

*Step 5.* Test the values of *C*_hs_ and η_hs_ while also allowing for changes in the value of λ from Step 3 against those used previously to determine if there is any significant change in *C*_hs_, η_hs_ and λ from Step 4. *C*_hs_ and η_hs_ are optimized using a box-constrained optimization algorithm from the statistics package available in R. Convergence is determined when there is no absolute change in *C*_hs_ of 0.1 or greater, no absolute change in η_hs_ of 0.1 or greater, or no order of magnitude change in λ. The process is run until the system has converged and then all values are passed to Step 6 (while loop).

*Step 6.* Reconstruct the cluster size distribution using optimized values for *C*_hs_, η_hs_ and λ from the magnetic scattering curve. From the size distribution, determine the size, number density and volume fraction of clusters with equations (17)–(20).

*Step 7.* Use the reconstructed cluster size distribution from the magnetic scattering to fit the nuclear and combined nuclear and magnetic scattering curves to determine the values for Δρ_nucl._, 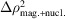 and the *A* ratio.

The computing procedure described above was verified using the simulated SAXS data from Pedersen (1994 ▸), including both the unimodal Gaussian peak distribution with η = 0.3 and *C*_hs_ = 1 and the bimodal Gaussian peak distribution with η = 0.1 and *C*_hs_ = 1.4422. Both cases included a statistical fluctuation of 3% in the simulated intensity. The fitted simulated data are provided in Fig. S1 in the supporting information. The size distribution recovered using the above algorithm is in line with that from the similar procedure proposed by Tsao *et al.* (1999 ▸) and exhibits a marked improvement over the original fitting performed as described by Pedersen (1994 ▸). The fitting given in Fig. S1 provides the necessary basis for the application of the computer routine to the analysis of small-angle scattering data, including those acquired using the SM-SANS technique.

The error analysis for the FF method which considers the covariances is completed using the Monte Carlo procedure proposed by Svergun & Pedersen (1994 ▸) and recommended for use by Pedersen (1997 ▸). In this procedure, 100 individual magnetic scattering curves are generated by randomly selecting a value at each *q* based on the mean and standard deviation of the individual intensity at each discreet *q* and then solving the unique set of coefficients to the basis functions while holding *C*_hs_, η_hs_ and λ constant. From the coefficients, the errors on the volume fraction, mean radius and number density are estimated as the standard error of the mean of the values based on the 100 iterations. In addition, the error in the size distribution is acquired from the Monte Carlo procedure and is reported as the 95% confidence interval over 0 to *R*_max_, where negative values are forced to zero for graphical representation. The standard error for *C*_hs_ and η_hs_, as well as the values obtained from the LN size distribution, are derived from the Hessian matrix of the PORT optimization (Gray, 1990 ▸) or the standard error reported directly from the Levenberg–Marquardt fitting routines, respectively. The standard errors in the nuclear scattering contrast and the combined nuclear and magnetic scattering contrast are extracted from the Levenberg–Marquardt fitting routines.

### 2.5. APT configuration and analysis

The data for the C35M and 125YF specimens have previously been presented elsewhere in the literature (Massey *et al.*, 2019*b* ▸; Zhang *et al.*, 2019 ▸). For all specimens, a CAMECA model 4000X HR local electrode atom probe (LEAP) at either the Center for Advanced Energy Studies (CAES) at Idaho National Laboratory or the Center for Nanophase Materials Sciences (CNMS) at ORNL was used to collect the required data. All specimens were run in laser mode with a pulse frequency of 200 kHz and a target detection rate of 0.005 atoms per pulse at a tip temperature of 50 K. A laser energy of 50 pJ was used for the C35M specimens, whereas a laser energy of 32 pJ was used for the 125YF specimens. All preliminary data analysis was performed using CAMECA’s *Integrated Visualization & Analysis Software* (*IVAS*, Version 3.6.8). Cluster identification and geometry were determined, based on the maximum separation method.

The reported number density of the observed clusters using APT was determined by
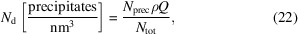
where *N*_prec_ is the number of precipitates as determined by the maximum separation method, ρ is the atomic volume of the material, *Q* is the detection efficiency (36% for LEAP 4000X HR) and *N*_tot_ is the total number of ions in the control volume. As a first-order approximation, the atomic volume of b.c.c. Fe at room temperature is used (ρ = 84.3 atoms nm^−3^). The error in the number density was determined from the standard deviation of the reported values for each sample group in a single condition. The radius of each cluster was derived from the radius of gyration *R*_g_,
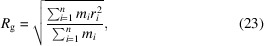
where *n* is the number of atoms in the precipitate, *m_i_* is the mass of an individual atom and *r_i_* is the distance of an individual atom from a cluster’s center of mass. Here, the radius of gyration is extracted through the maximum separation method output in *IVAS*. Although the radius of gyration is a value that has physical context towards the cluster radius, it is typically perceived that the value derived from APT underestimates the size of the cluster. The radius of spherical equivalent (*R*_s_, also denoted as the Guinier radius) is a more appropriate descriptor of the cluster’s physical dimensions (Prakash Kolli & Seidman, 2007 ▸). *R*_s_ was calculated simply from *R*_g_ using
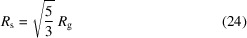
when the cluster is assumed to exhibit a spherical shape. Another method, denoted here as the radius of atomic count *R*_a_, can also be used as means of describing the cluster radius from APT data. The radius of atomic count considers each cluster to be spherical and can be calculated using
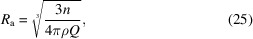
where equation (25) shares the same variable definitions and values as equation (22), except here *n* denotes the number of atoms associated within an individual cluster. Again, as a first-order approximation the atomic volume of b.c.c. Fe is used. This assumption, as discussed later in detail, is valid for clusters which exhibit a similar structure to the host matrix, but the assumption introduces error when the cluster structure deviates significantly from that of the matrix (*e.g.* b.c.c. Fe in the cases presented herein).

Prior studies that sought to compare the cluster dimensions and number density derived from APT analysis with those of SANS analysis (Hyde *et al.*, 2014 ▸; Briggs *et al.*, 2017 ▸; Simm *et al.*, 2017 ▸; Cunningham *et al.*, 2014 ▸) typically used the mean of the calculated values directly from equations (22)–(25) for comparisons. Such approaches fail to consider both the resolution and constraint of the SANS configuration and the analysis technique, respectively. In addition, the approach ultimately assumes that the size distribution reported from APT is normally distributed. To provide a more accurate comparison between techniques, a size distribution for *R*_g_, *R*_s_ and *R*_a_ was first derived from the APT data by generating a binned histogram with the same spatial point resolution, and thus number of basis functions *N*_SANS_, as the size distributions developed in the SANS technique to calculate 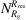, 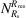 and 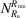 for each condition. These size distributions were then fitted using equation (13), where *N* in equation (13) was equal to the number of cubic spline basis functions used in the SANS analysis from Table 2 ▸ and equation (14). An additional evaluation was completed with *N* = 2*N*_SANS_, thus enabling a determination of the variance between APT- and SANS-derived radii when using the common approach, an approach which considers the resolution limitations of SANS (*N* = *N*_SANS_), and if the SANS resolution was increased significantly (*N* = 2*N*_SANS_). The three separate size distributions for each APT-derived radius were used to extract the values for the mean radius, volume fraction and number density of clusters using the moments of the size distributions and equations (17)–(20). Errors using the described procedure were determined using the same methods as described above for the SANS analysis.

## 3. Results and discussion

### 3.1. Two-dimensional scattering patterns

**Figure 3:**
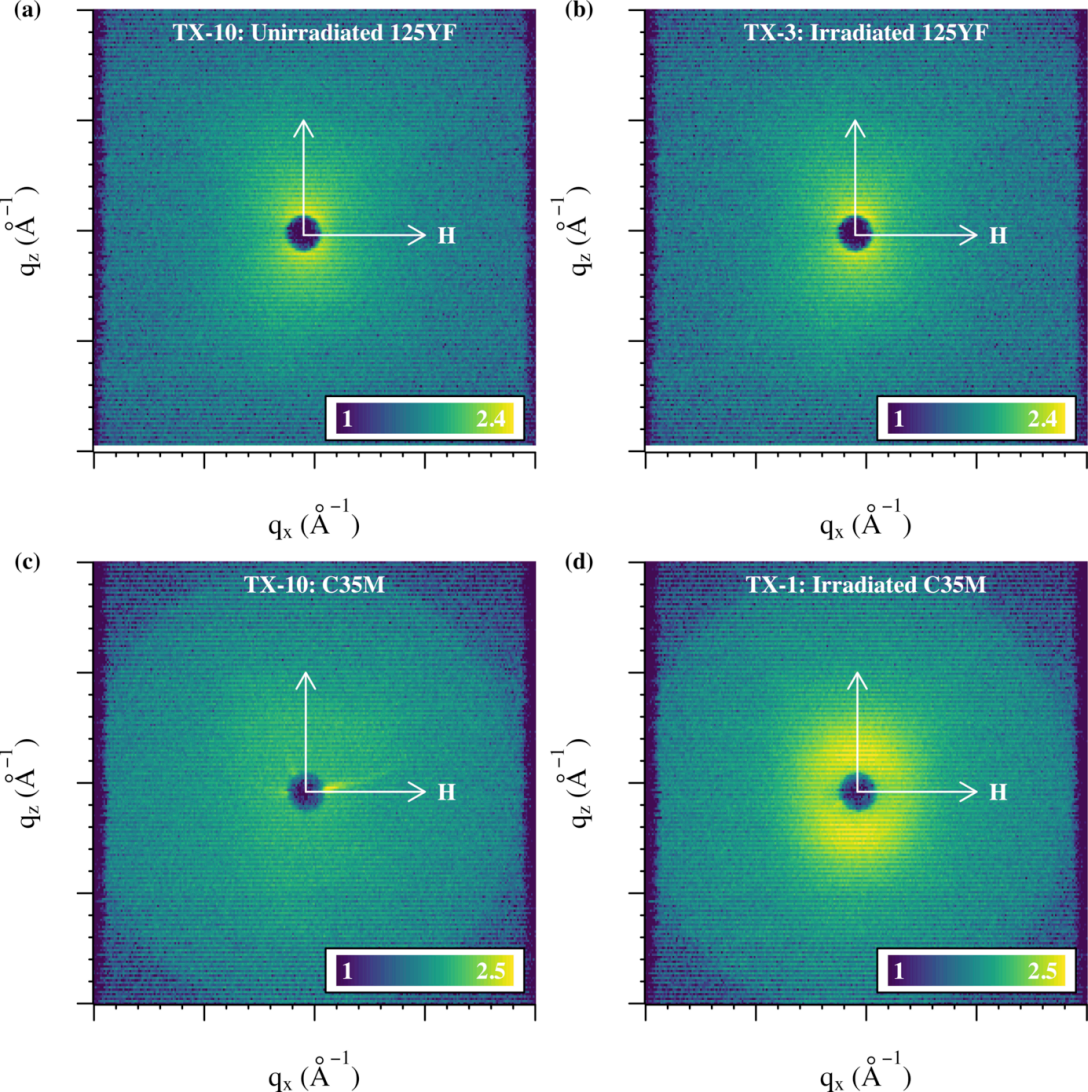
Two-dimensional scattering patterns (neutron counts) for (*a*) 125YF specimen in the as-received state, (*b*) 125YF specimen after irradiation to 1.8 dpa at 357°C, (*c*) C35M specimen in the as-received state and (*d*) C35M specimen after irradiation to 1.8 dpa at 357°C. 125YF data from 0.8 nm neutrons at a sample-to-detector distance of 1.5 m. C35M data from 0.6 nm neutrons at a sample-to-detector distance of 1.2 m. Color bar represents the log of total counts.

The 2D scattering patterns can provide insight into the quality of the shielding components, as well as the influence of the magnetic field on the scattering of the clusters. Fig. 3 ▸ shows examples of the 2D SM-SANS patterns obtained from the saturating magnetic field for both the 125YF and C35M specimens in the as-received state and the irradiated state. The 125YF patterns show an anisotropic pattern in Figs. 3 ▸(*a*) and 3 ▸(*b*), where the scattering aligned to the magnetic field (nuclear scattering) shows a reduced intensity compared with the intensities perpendicular to the magnetic field. A similar but more intense variance was observed for the irradiated C35M specimen, as shown in Fig. 3 ▸(*d*). This anisotropic nature indicates paramagnetic scatterers within a ferromagnetic matrix and enables details of the composition of the scattering par­ticles to be derived from the *A*-ratio calculation in equation (21).

The as-received C35M specimen presented in Fig. 3 ▸(*c*) shows a nearly isotropic pattern near background levels. The general pattern indicates limited clustering or precipitation in the system, as expected from *a priori* knowledge. Interestingly, Fig. 3 ▸(*c*) does show the presence of three streaks in various orientations in the pattern. A similar but less obvious streak can be seen in Fig. 3 ▸(*d*) at the eight o’clock position near the detector center. These streaks were identified as artifacts from the Pb shielding used in the SM-SANS configuration. Pixel masking was used on the 2D scattering patterns to eliminate artifacts arising from the Pb shielding. These artifacts can also be eliminated by using an incident wavelength of 0.8 nm, which is beyond the Bragg cutoff for Pb, but this would reduce the maximum accessible *q* and hence the minimum measurable precipitate size.

### 3.2. Measurement of precipitate size distributions

**Figure 4:**
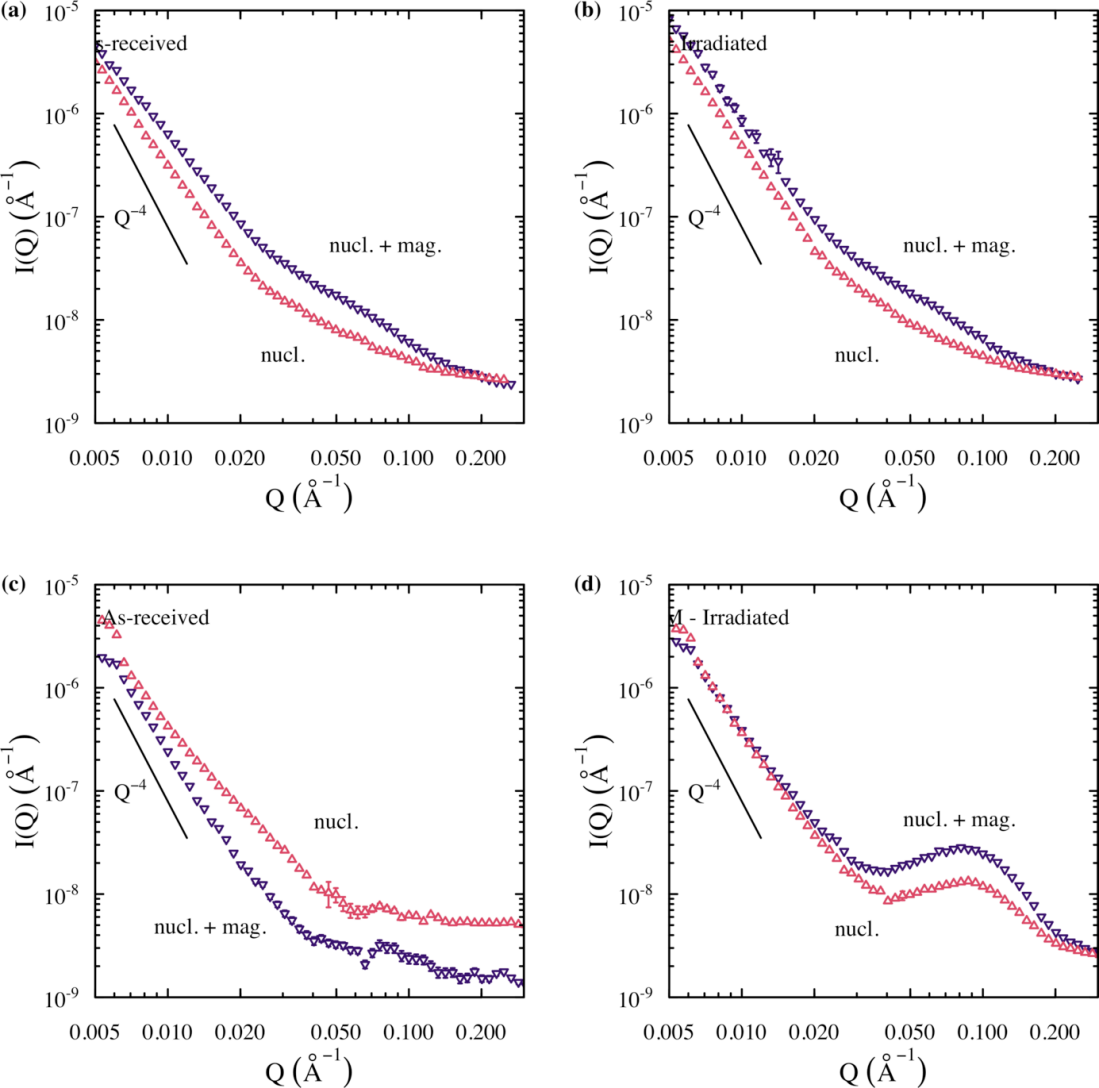
Scattering intensity perpendicular to the magnetic field (nucl. + mag.) and parallel to the magnetic field (nucl.) determined using SM-SANS for (*a*) 125YF specimen in the as-received state, (*b*) 125YF specimen after irradiation to 1.8 dpa at 357°C, (*c*) C35M specimen in the as-received state and (*d*) C35M specimen after irradiation to 1.8 dpa at 357°C.

**Figure 5:**
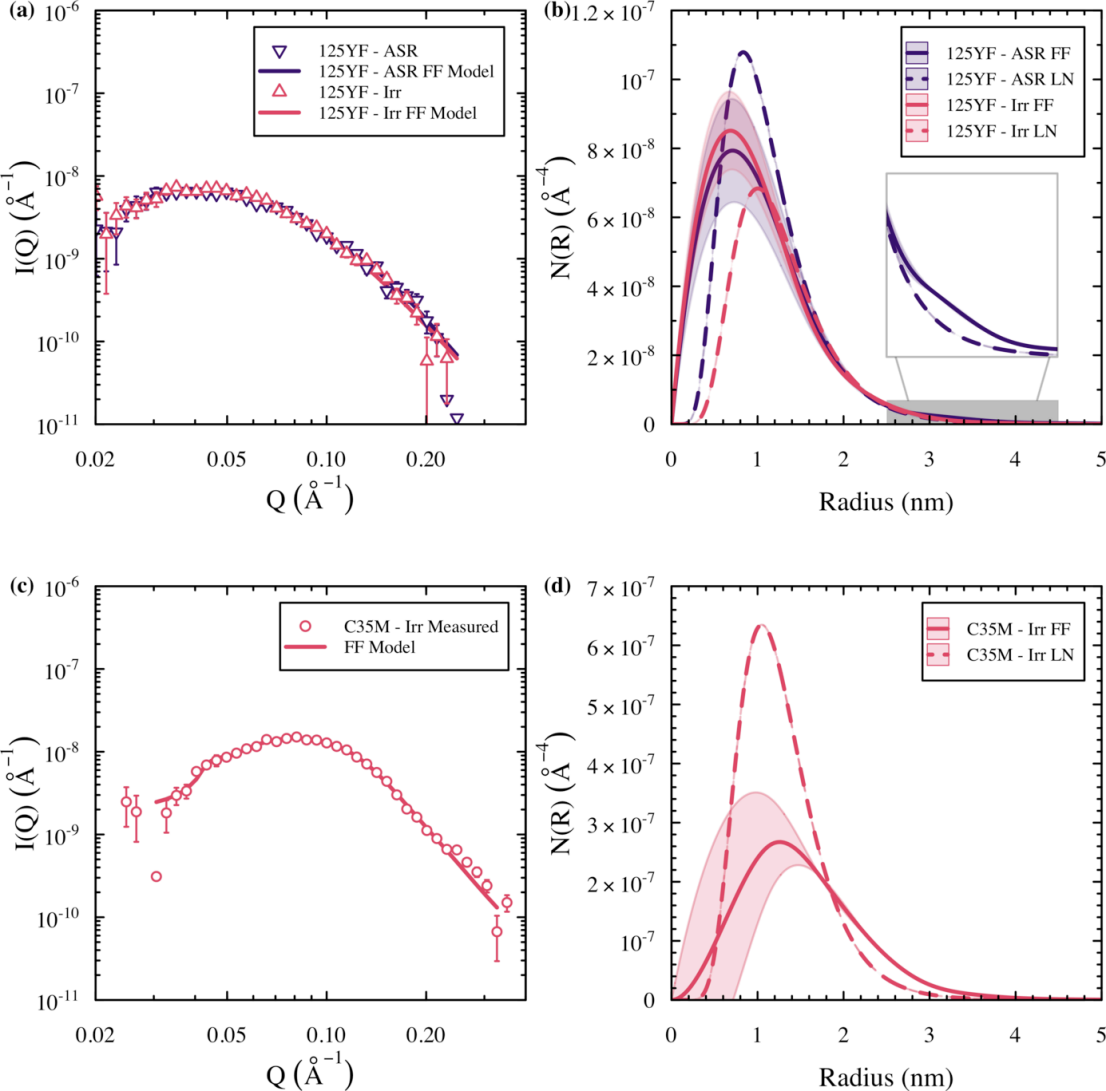
(*a*) and (*c*) Magnetic scattering intensity (points) and FF local monodisperse approximation model (lines), with (*b*) and (*d*) the corresponding LN and FF size distributions, for the 125YF and C35M specimens. The shaded areas in the size distributions represent the 95% confidence interval from Monte Carlo-based error analysis. The gray rectangle in panel (*b*) indicates the scale region of the inset showing the small secondary peak at 3.2 nm in the FF size distribution.

After pixel masking in the 2D scattering patterns across the three detector configurations, the data were reduced and combined to form 1D scattering curves in the direction parallel to the magnetic field (horizontal in the page in Fig. 3 ▸) and perpendicular to the magnetic field. This configuration extracts the nuclear scattering cross section and the combined nuclear and magnetic scattering cross sections across the *q* range of interest, as shown in Fig. 4 ▸. The peaks observed in the *q* range of 0.020 to 0.2 Å^−1^ are indicative of scattering as the result of nanometre-sized clusters in the matrix. Interestingly, Fig. 4 ▸(*c*) shows the nuclear scattering cross section to be higher than that of the combined scattering for the unirradiated C35M alloy. This artifact was caused by the streaks seen in Fig. 3 ▸(*c*) that were not fully masked during the data reduction procedure, highlighting the critical need for ultra-high-purity Pb shields in future variants, as well as detailed evaluation of both the 2D and 1D data sets by domain experts. Because of this artifact and the knowledge that no clusters were present in the material in the unirradiated state (Gussev *et al.*, 2017*a* ▸; Yamamoto *et al.*, 2015 ▸), the data in Fig. 3 ▸(*c*) were not analyzed in detail and are omitted from further presentation herein. The data presented in Fig. 4 ▸ were used to estimate the magnetic scattering of the clusters and the estimation was then used to determine the precipitate size distributions from the LN and FF approaches of the remaining data sets. The result­ing model fits and size distributions are presented in Fig. 5 ▸.

Fig. 5 ▸ shows good agreement between the FF model and the experimentally determined magnetic scattering cross sections within the *q* range of interest using the inputs provided in Table 2 ▸. The differences in the size distributions between the LN and FF approaches in Fig. 5 ▸ are minimal, but an important difference can be observed. For instance, in the case of the unirradiated 125YF specimen, the FF size distribution shows a small secondary peak (<5 × 10^−9^ Å^−4^) in the size distributions centered around 3.2 nm that is not effectively captured using the LN approach. Similar bimodal distributions have been captured using a summation of two log-normal distributions in an Fe–10Cr–Al alloy with Y–Al–O clustering (Massey *et al.*, 2019*a* ▸). This bimodal size distribution was captured intrinsically here using the FF approach. After irradiation, the peak at 3 nm in the size distributions is reduced, suggesting that larger clusters are unstable due to ballistic dissolution dominating the clusters’ radiation response (Certain *et al.*, 2010 ▸; Wharry *et al.*, 2017 ▸). The radiation stability of the Y–Al–O nano-clusters will be the focus of an additional follow-on study, as it requires more irradiation conditions and additional complementary techniques such as energy-filtered TEM.

### 3.3. Measurement and evaluation of microstructural parameters

The microstructural parameters, including mean radius and number density, from both the LN and FF approaches using the SM-SANS data are provided in Table 3 ▸, while the fitted hard-sphere interaction parameters, including the volume fractions from the FF technique, are given in Table S3. Not surprisingly, according to the size distributions in Figs. 5 ▸(*b*) and 5 ▸(*d*), the values from the LN and FF approaches are generally within 10 to 20% of each other for both 125YF specimens and the irradiated C35M specimen. No quantification was performed on the as-received C35M specimen because the 2D and 1D scattering did not provide viable indications of clusters in the specimen. The SM-SANS-based observation of no observed clustering in the as-received C35M specimen was consistent with the APT observations performed on the same specimen.

The full list of derived microstructural parameters based on the APT analysis is provided in Table S2 in the supporting information. In general, little variation in the microstructural parameters is observed in Table S2 for *R*_s_, *R*_a_ and *R*_g_, even when the number of basis functions is doubled, thus indicating that increased resolution in the SANS measurements would not be likely to improve the overall agreement between the APT and SANS data sets. Increasing the resolution of the fitted size distribution via SANS even further could be considered, but increasing the number of cubic spline basis functions in the FF approach results in overfitting and numerical instabilities with the SANS-based data. Although such observations are worth evaluating, they are mostly irrelevant to the comparison between the APT and SANS results for the materials systems studied.

Table 3 ▸ presents the values of the mean radius obtained using the spherical equivalent (*R*_s_) and the atomic count method (*R*_a_) from the APT analysis to compare directly with the cluster radius *R*_p_ from SANS experiments. Although the radius of gyration *R*_g_ is reported in Table S2, it is excluded from continued discussions given the recommendations of Prakash Kolli & Seidman (2007 ▸), who indicate favoring the spherical equivalent when comparing the physical dimensions of a precipitate via APT. Also, note that the values from the APT analysis in Table 3 ▸ are calculated by fitting the APT size distributions with same number of basis functions as the SANS FF approach. In the case of the two 125YF specimens, the SANS approaches (FF and LN) do not align with the mean radii reported using the atomic count method from the APT analysis. In contrast, the values from the SANS approach for the irradiated C35M alloy show only a 0.1 nm variance in the reported radii, which can be considered within the error of the sampling and calculation techniques.

The disagreement between the reported radii for the APT-based atomic count method and SM-SANS FF analysis is not unexpected for the 125YF specimens. Here, we take the basis that no *a priori* knowledge of the cluster structure exists and thus the atomic volume is assumed to be equivalent to b.c.c. Fe. Previous reports suggest that a range of different phases for the Y–Al–O clusters are known to exist in 125YF specimens (Dryepondt *et al.*, 2018 ▸; Massey *et al.*, 2019*a* ▸). These include yttria alumina garnet (YAG), yttria alumina perov­skite (YAP) and yttria aluminium monoclinic (YAM), and other possible phases including yttria (Y_2_O_3_), alumina (Al_2_O_3_) and aluminium nitride (AlN). All of these phases could exist with various volume fractions in the matrix and each could exist with different atomic volumes, and thus they could alter the calculation of the cluster radii via the atomic count method. For example, YAG has an atomic volume of 93 atoms nm^−3^, whereas YAM’s is significantly less at 74 atoms nm^−3^ (Yamane *et al.*, 1995 ▸). Applying these values to equation (25) results in a ∼10% variance in the mean value for *R*_a_, which still does not reach an alignment with the SM-SANS FF values reported in Table 3 ▸, but it does show that uncertainties in the structure of the scatterers can contribute to variances between reported SANS and APT values. This effect highlights the deficiencies associated with the APT-based atomic count analysis method and the need to execute complementary experiments to ascertain likely bounds in the nanoscale precipitation characteristic of alloys with uncertain or highly varying precipitate structures.

**Figure 6:**
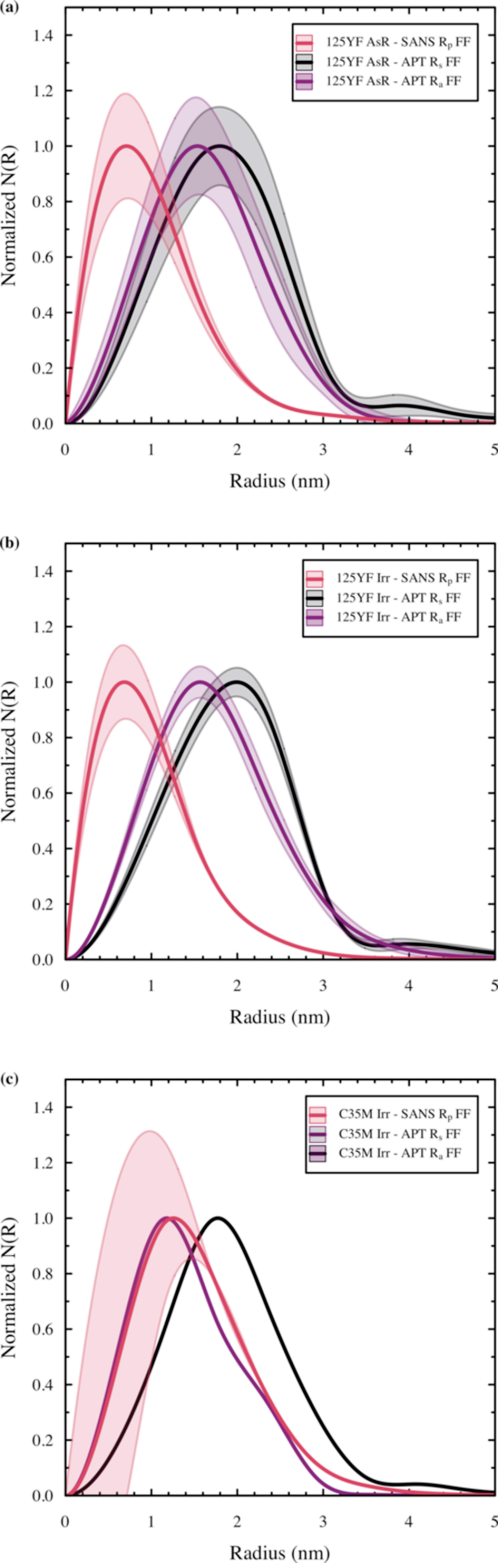
Comparison of SANS-fitted FF local monodisperse approximation size distributions with APT-derived fitted FF size distributions for *R*_s_ and *R*_a_ for all scatterers when the numbers of basis functions are identical between the two techniques for (*a*) 125YF specimen in the as-received state, (*b*) 125YF specimen after irradiation to 1.8 dpa at 357°C and (*c*) C35M specimen after irradiation to 1.8 dpa at 357°C. Shaded areas in size distributions represent the 95% confidence interval from Monte Carlo-based error analysis.

The SANS mean radius and the radius of spherical equivalent also appear to have discrepancies for the 125YF specimens. The disagreement is observed in Table 3 ▸ and it is also seen when directly assessing the normalized size distributions compared with the mean values shown in Figs. 6 ▸(*a*) and 6 ▸(*b*). The primary peak from the SM-SANS FF approach is consistently lower than the *R*_s_ peak from the APT analysis in both unirradiated and irradiated conditions for the 125YF specimens. As indicated by APT, the addition of Cr-rich clustering in the irradiated specimen did shift the number density distribution reported for the SANS data, but it does not have a strong effect on the size distributions in the APT analysis because of the low volume fraction of detected Cr-rich clusters in each sample. The inconsistencies between *R*_s_ and the SM-SANS FF approach imply that additional experimental artifacts could be at play, including the aforementioned trajectory aberration effects present in the APT technique, especially when two phases with dissimilar properties are present such as are seen in the 125YF samples studied herein (Gault *et al.*, 2012 ▸; Hyde *et al.*, 2014 ▸; Hatzoglou *et al.*, 2019 ▸).

Ignoring the mean values and focusing on the shape characteristics alone in Figs. 6 ▸(*a*) and 6 ▸(*b*), the observed difference in the size distributions for the 125YF samples, including the extended tails seen in Figs. 6 ▸(*a*) and 6 ▸(*b*), most likely arises from heterogeneity of the clusters in the matrix. The 125YF alloy is a powder-metallurgy-derived alloy and it is well known that the processing techniques used to produce the clustering impart a heterogenous distribution of clusters, including the precipitation of larger clusters along the grain boundaries (Dryepondt *et al.*, 2018 ▸). The SM-SANS technique samples orders of magnitude larger volumes, which effectively measures both the smaller intra- and larger inter-granular clustering. The low sampling volume (∼10^−21^ m^−3^) of the APT technique means that only one grain or a few grains are sampled, so the results are strongly biased toward intragranular clustering. Significantly larger APT sampling is needed to confirm that the tailing observed in the SANS-derived size distributions results from increased cluster sizes on the grain boundaries.

Compared with the 125YF specimens, the irradiated C35M specimen shows stronger agreement between the mean radius obtained using the atomic count approach for APT and that from the SM-SANS FF approach (Table 3 ▸). This is also true for the size distributions [Fig. 6 ▸(*c*)], where the SANS-extracted size distribution better approximates the shape, including the skewness and FWHM, of the size distribution extracted from the atomic count approach. This is expected because the Cr-rich clusters are known to have atomic volumes that approximate that of the host matrix (Ribis & Lozano-Perez, 2012 ▸), providing more validity in the use of equation (25) and its associated simplifying assumptions.

Additional variation is expected in the shape of the size distributions between the SM-SANS-based approaches and the APT-based approach, primarily resulting from the inherent artifacts associated with each technique. For instance, the scattering intensity is proportional to *r*^6^ when *q* approaches zero (Sequeira *et al.*, 1995 ▸). The result is that the reconstructed size distribution is poorly defined for smaller clusters (*e.g.* <1–2 nm) and is usually a simple extrapolation to zero when the radius approaches zero. This effect can be seen in the increased uncertainty resulting from the Monte Carlo error analysis below the local maximum peak in the size distributions presented in Figs. 5 ▸(*b*) and 5 ▸(*d*). Therefore, some uncertainty exists in the size distribution and the resulting mean radius, particularly when clusters are below 1 nm. Similar concerns arise in sizes derived from APT reconstructions such as APT-specific lateral resolution and trajectory aberration artifacts (Hyde *et al.*, 2014 ▸; Briggs *et al.*, 2017 ▸). It has been demonstrated that the 0.85 nm lateral resolution of the LEAP 4000X HR used in this work, coupled with the low detection efficiency (36%) of the unit, results in 11–14% of matrix atoms entering Cr-rich precipitates in similar compositions to those seen in this work for C35M (Hatzoglou *et al.*, 2019 ▸). The slight difference in local evaporation fields for Fe and Cr also results in trajectory aberrations that cause an artificial compression in the lateral direction of the APT analysis with an elongation in the depth direction of analysis. Fortunately, however, these largely offset for the Cr-rich precipitates and no significant differences in simulated versus measured precipitate radii were reported when the Guinier radius was used (Hatzoglou *et al.*, 2019 ▸). For the 125YF specimens, the evaporation field differences are much greater between the Y–Al–O-rich particles and the FeCrAl matrix, which is likely to cause more significant ion trajectory focusing effects and further variations in APT precipitate data sets.

Table 3 ▸ shows good agreement in the number density between the SANS and APT techniques for the 125YF specimens, with reported values a factor of two from each other within the same order of magnitude. In the case of the C35M specimen, the number densities are within the error of each other with a variance close to 30% (Table 3 ▸). The absolute magnitude of the size distribution in SANS is dependent on the estimated scattering contrast. The scattering contrasts 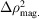 are estimated from the bulk atomic fraction of Cr and Al contents (Section 2.3). This estimation does not account for the reduced concentration of Cr and/or Al in the matrix because of the Cr clustering that occurs in the irradiated C35M and 125YF alloys (*e.g.* Cr being sequestered at grain boundaries or within the Y–Al–O clusters in the unirradiated 125YF specimens) or contributions of minor alloy elements such as Mo in the C35M specimens to the scattering contrast.

In the case of the 125YF specimens, these factors appear to have an effect, as indicated by the variance seen in Table 3 ▸. In the C35M specimen, these effects could be significant when estimating the 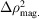 value in Table 2 ▸ and the true values for the investigated specimens. The addition of Mo in the C35M specimen compared with the 125YF specimens could significantly alter the mean magnetic moment of the alloy, but at the time of writing the magnitude of such an effect is unknown.

An additional consideration is the assumption that the mean magnetic scattering length in the clusters is effectively zero. Studies of the Fe–Cr system (Fallot, 1936 ▸; Aldred, 1976 ▸) have shown that the magnetic moment of the Fe–Cr system decreases approximately linearly with increasing Cr and effectively becomes zero when *x*(Cr) > 0.85. This effect is captured using Blau’s empirical method and the Al effect is also extrapolated. In the composition range of 0.0 < *x*(Cr) < 0.8 and 0.0 < *x*(Al) < 0.2, all clusters are anticipated to exhibit a magnetic moment. Altering the prior assumption that the magnetic moment of the clusters is zero to include a magnetic moment for the clusters would effectively decrease 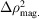 based on equation (4). The amount of the decrease is directly related to the assumed concentration of Cr and Al in the clusters. Further discussions on composition considerations are reserved for the following section, but any decrease in 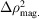 results in a direct change in the number density. For instance, when 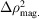 is set to a theoretical value of 1.10 × 10^−11^ Å^−4^, the number density of the clusters was found to be 40.7 (0.6) × 10^−23^ m^−3^. Even in this rough form, the analysis suggests that in fact the largest discrepancy in the error associated with the number density of scatterers in the C35M specimen using the SM-SANS FF technique arises from the calculation of the mean magnetic moment of the matrix – such as the effect of Mo – and not the assumption that the scatterers are antiferromagnetic.

An attempt was made to optimize the estimated 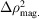 values by driving the size-distribution-derived values closer to those from equation (3) using an optimization step, but this attempt proved to be inconsistent because of the numerous local minima in the parameter optimization space for η_hs_ and *C*_hs_. These local minima also vary depending on the estimated 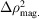, resulting in a circular function with limited ability to converge. Therefore, the attempt to more closely correlate the values determined using equations (3) and (20) was abandoned because the uncertainty in the estimate of 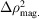 could not be improved without further inputs such as detailed matrix/cluster compositions from APT.

Ultimately, the deviations between the SM-SANS values and APT values given in Table 3 ▸ are intrinsic to comparing any small-angle scattering technique with APT and are not a development from using the Pb-shielding approach proposed herein. The presented results indicate that the SM-SANS technique is a readily viable approach to obtain useful information regarding the size distribution and microstructural parameters of nanometre-sized precipitates and clusters in highly radioactive specimens. The unique factor that must be considered for the SM-SANS technique is the artifacts in the 2D scattering pattern from the Pb shielding. This is controlled by simple pixel masks in the 2D scattering patterns prior to data reduction. The greatest uncertainty arises from the estimation of the magnetic scattering contrast of the investigated system, which has the highest impact on the values for volume fraction and number density of the scattering clusters.

The FF approach proposed herein, which is based on the approach of Pedersen (1994 ▸), also enables a means of extracting a higher level of fidelity in the size distribution compared with more simplistic approximations such as the LN approach. In both cases, the LN and FF approaches yield values that provide direct correspondence to the APT approach. The result is that the SM-SANS technique coupled with the FF approach is an effective means of performing correlative studies of nanoscale clustering in irradiated material systems which have effective scattering contrast within a neutron beam.

### 3.4. Measurement and evaluation of composition and structure parameters

The *A* ratios derived from the SM-SANS technique provide the ability to evaluate the composition and structure of the scatterers. Table 4 ▸ provides the fitted scattering length densities and *A* ratios. For the 125YF unirradiated sample, the *A*-ratio value is abnormally low for the probable phases for the dominant Y–Al–O nano-clusters. For example, the YAG, YAP and YAM phases in the 125YF matrix are expected to have *A* ratios of 5.9, 11.0 and 3.6, respectively. Although the *A* ratio does not match any of the theoretical values, the value of 2.60 ± 0.06 is close to the values reported for FeCrAl alloys with Y–Al–O additions (Massey *et al.*, 2019*a* ▸). For brevity, the reader is referred to the extensive discussions by Massey *et al.* (2019*a* ▸) on the interpretation of the reported *A* ratios in comparison with the theoretical values. Simply, the reduced *A* ratio compared with the theoretical values suggests that the Y–Al–O clusters are non-stoichiometric and/or highly defective, but this does not allow for direct conclusions on the phase(s) of the Y–Al–O clusters (Massey *et al.*, 2019*a* ▸). The conclusions of Massey *et al*. are supported within this work by the inconsistencies found between the APT and SM-SANS techniques, as discussed in the previous section.

**Figure 7:**
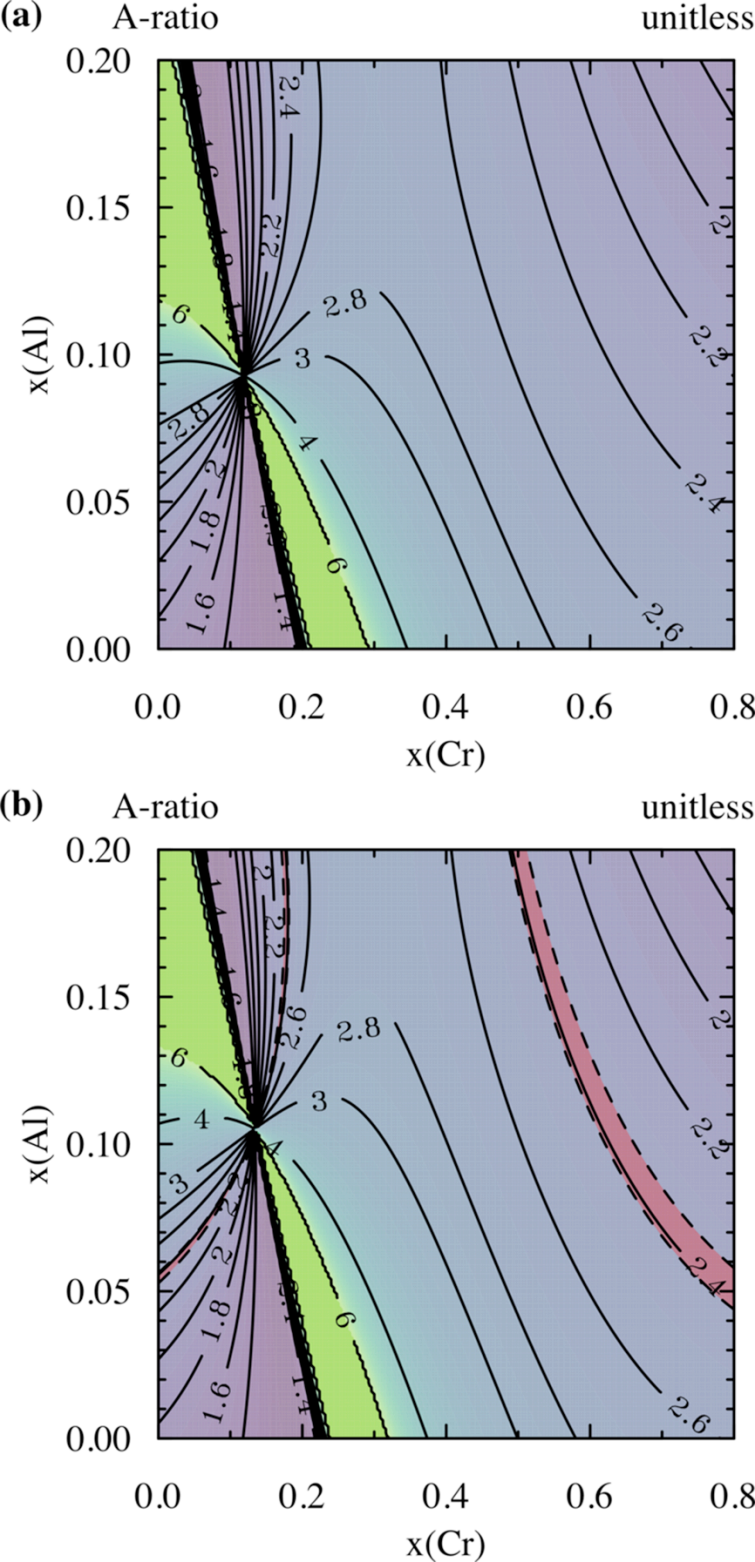
Calculated *A* ratio of the α′ phase as a function of Cr and Al contents in a nominal (*a*) 125YF and (*b*) C35M matrix. The red shaded area in panel (*b*) represents the possible composition range based on the fitted *A*-ratio value for the irradiated C35M specimen from the SANS-fitted FF local monodisperse approximation size distributions (Table 4).

More interesting, and unique to the SM-SANS technique, is the observation of a reduction in the *A* ratio for 125YF after irradiation. Previous APT analysis (Massey *et al.*, 2019*b* ▸) has shown that Cr-rich α′ forms after 1.8 dpa irradiation at 357°C in 125YF. The formation of this secondary scatterer results in a change in the nuclear and magnetic scattering length densities and thus the *A* ratio. This is observed in the case of the fitted nuclear scattering length densities, as seen in Table 4 ▸. The magnitude of these changes, as discussed in Section 2.3, is directly linked to the Cr and Al contents in the Cr-rich α′ clusters. This has been calculated for the 125YF specimen and is provided in Fig. 7 ▸(*a*), taking into account the contribution of Fe to the magnetic scattering contrast of the matrix and the Cr-rich α′ clusters.

On the basis of the previous APT analysis of the cluster composition, where the Cr and Al contents were determined to be 60.72 ± 10.67 and 5.37 ± 2.96 at.% (Massey *et al.*, 2019*b* ▸), the minimum and maximum theoretical values from only the Cr-rich α′ are 2.33 and 2.88, respectively. The value of 2.33 is associated with an expected composition rich in Cr, whereas the opposite is true for the *A* ratio of 2.88, as seen in the contour plot in Fig. 7 ▸(*a*). The shift in the *A* ratio from 2.60 ± 0.06 to 2.44 ± 0.06 after irradiation confirms the APT observations of dual-phase formation under irradiation (Massey *et al.*, 2019*b* ▸) and suggests that the α′ that has formed under irradiation is rich in both Cr and Al. The exact composition from SANS is impossible to deconvolute because the composition/structure of the Y–Al–O clusters could also have evolved under irradiation and the volume fraction of the Y–Al–O clusters and Cr-rich α′ cannot be determined from the SM-SANS technique alone.

The irradiated C35M specimen could provide additional information regarding the general composition of the Cr-rich α′ formed in irradiated FeCrAl because the sample is devoid of Y–Ti–O clusters. The determined *A* ratio from the SM-SANS FF technique was 2.38 ± 0.03, with the same fit to the nuclear scattering contrast as the irradiated 125YF specimen (Table 4 ▸). Again, the *A* ratio will vary depending on the Cr and Al contents because of the variances in both the nuclear and magnetic scattering contrast. Fig. 7 ▸(*b*) displays the variance with changing Cr and Al contents in the C35M irradiated specimen. Fig. 7 ▸(*b*) shows that the experimentally determined *A* ratio is associated with Cr- and Al-rich α′, suggesting a composition similar to that expected in the 125YF specimen. These observations are consistent with recent studies on irradiated FeCrAl alloys (Briggs *et al.*, 2017 ▸; Massey *et al.*, 2019*b* ▸; Zhang *et al.*, 2019 ▸; Edmondson *et al.*, 2016 ▸), although those studies indicate that the Cr content can vary according to the irradiation condition and the starting matrix composition. Furthermore, the reported *A* ratio for the C35M specimen is consistent with the ranges reported for Cr-rich α′ in irradiated Fe–Cr alloys (Mathon *et al.*, 2003 ▸; Reese *et al.*, 2018 ▸; Bachhav *et al.*, 2014*a* ▸, 2014*b* ▸).

The application of the *A* ratio towards deconvoluting the composition of the α′ clusters is complicated as a result of the necessary theoretical values for calculating input factors, including the atomic volume of the matrix and the precipitate, as well as the mean magnetic moment of both scattering features. Here, empirical fitting is used in both instances to calculate these input values. In both instances, fits were completed (Preston, 1932 ▸; Taylor & Jones, 1958 ▸; Blau *et al.*, 1977 ▸) only for data in the Fe-rich corner of the Fe–Cr–Al system. Thus, the largest uncertainty exists in the *A* ratio determination when extrapolating these relationships into the Cr-rich corner of the Fe–Cr–Al system. Until more empirical or modeling data are derived for the typical composition ranges expected for α′ in irradiated Fe–Cr alloys, the analysis will be limited in extending past the inference methods discussed herein. These limitations are not inherent to the proposed SM-SANS technique; they also extend to unshielded magnetic SANS techniques. At a minimum, it can be determined from the analysis that the Pb shielding in the SM-SANS configuration does not affect the ability of the SM-SANS technique to interrogate first-order compositional and structural changes in scattering clusters.

## 4. Conclusion

The SM-SANS technique was explored for as-received and irradiated FeCrAl alloys, C35M and 125YF. The 2D scattering patterns show that artifacts can arise from the Pb shielding used in the experimental configuration of the SM-SANS techniques. Pb-shielding-based artifacts are readily removed using simple pixel masking techniques, thus allowing for detailed quantitative analysis of microstructural parameters of clustering in these two FeCrAl alloys. Comparison of the SM-SANS results and the APT results shows that SM-SANS can effectively characterize nanoscale clustering and precipitation in the alloys, and the application of a magnetic field also provides insights into the compositions of the scattering clusters/precipitates. Even in this first iteration, the work shows that shielded SANS is an effective means of nanoscale characterization of clustering and precipitation in radioactive nuclear materials specimens.

## Supplementary Material

Additional tables and figures. DOI: 10.1107/S1600576725003176/iu5075sup1.pdf

## Figures and Tables

**Table 1:** Bulk compositions (at.%) of alloys 125YF and C35M3 in the as-received state

Alloy	Fe	Cr	Al	Mo	Si	Y	O	C	N
125YF	Balance	11.77	9.19	0.01	0.02	0.11	0.63	0.09	0.08
C35M3	Balance	13.24	10.38	1.10	0.24	0.03	0.004	0.001	0.004

**Table 2:** Input parameters for the unconstrained local monodisperse approximation fitting model to the 1D SM-SANS data N.M. stands for not measured.

Alloy	Condition	Fit range (Å^−1^)	Δ*R* (Å)	*R*_max_ (Å)	*N*_SANS_	*a*_0_ (Å)	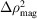 (× 10^−11^ Å^−4^)
125YF	As-received	0.0266 ≤ *q* ≤ 0.2477	12.7	100	12	2.88	1.62
	Irradiated	0.0266 ≤ *q* ≤ 0.2477	12.7	100	12	2.88	1.62
C35M3	As-received	N.M.	N.M.	N.M.	N.M.	N.M.	N.M.
	Irradiated	0.0305 ≤ *q* ≤ 0.327	9.6	70	11	2.88	1.49

**Table 3:** Comparison of cluster characteristics for 125YF and C35M3 before and after irradiation as determined using FF and LN fitting of magnetic shielded SANS and APT Values in parentheses are the error for the respective technique. N.O. stands for not observed.

Alloy	Condition	Technique	Cluster type	R_p_ (nm)	*N*_p_ × 10^23^ (m^−3^)
125YF	As-received	APT *R*_s_†	Y, Al, O	1.9 (0.1)	6.9 (0.9)
		APT *R*_a_†	Y, Al, O	1.6 (0.1)	6.3 (0.9)
		SM-SANS LN	Y, Al, O	1.1 (0.0)	10.2 (6.0)
		SM-SANS FF	Y, Al, O	1.0 (0.2)	11.0 (0.9)

	Irradiated	APT *R*_s_†	Y, Al, O	2.0 (0.0)	6.1 (0.3)
			Cr-rich	1.8 (0.1)	0.6 (0.1)
			Y, Al, O + Cr-rich	2.0 (0.0)	6.7 (0.3)
		APT *R*_a_†	Y, Al, O	1.8 (0.0)	5.6 (0.3)
			Cr-rich	1.7 (0.0)	0.7 (0.1)
			Y, Al, O + Cr-rich	1.8 (0.0)	6.2 (0.3)
		SM-SANS LN	Y, Al, O + Cr-rich	1.3 (0.0)	7.6 (1.4)
		SM-SANS FF	Y, Al, O + Cr-rich	1.0 (0.1)	11.4 (0.6)

C35M	As-received	Any technique	Cr-rich	N.O.	N.O.

	Irradiated	APT *R*_s_†	Cr-rich	1.9 (0.0)	30.3 (1.6)
		APT *R*_a_†		1.4 (0.0)	30.8 (2.3)
		SM-SANS LN	Cr-rich	1.3 (0.0)	64.6 (14.4)
		SM-SANS FF	Cr-rich	1.5 (0.1)	42.8 (8.4)

† Values derived from fitting cubic spline basis functions where the number of functions is equal to the number of splines used in the FF fitting of the size distributions in the SANS data. Full analysis of APT-derived cluster characteristics is provided in Table S2 in the supporting information.

**Table 4:** Calculated scattering length densities and *A* ratios using the FF fitting technique on the magnetic shielded SANS data Values in parentheses are the errors for the respective techniques.

Alloy	Condition	Type	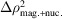 (× 10^−11^ Å^−4^)	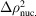 (× 10^−11^ Å^−4^)	*A* ratio
125YF	As-received	Y, Al, O	2.62 (0.03)	1.00 (0.02)	2.60 (0.07)
	Irradiated	Y, Al, O + Cr-rich	2.70 (0.04)	1.10 (0.02)	2.51 (0.07)

C35M	Irradiated	Cr-rich	2.58 (0.02)	1.08 (0.01)	2.38 (0.03)

## References

[bb1] Abrahamson, E. P. & Lopata, S. L. (1966). *Trans. Metall. Soc. AIME* **236**, 76–87.

[bb2] Aldred, A. T. (1976). *Phys. Rev. B* **14**, 219–227.

[bb3] Bachhav, M., Robert Odette, G. & Marquis, E. A. (2014*a*). *Scr. Mater.* **74**, 48–51.

[bb4] Bachhav, M., Robert Odette, G. & Marquis, E. A. (2014*b*). *J. Nucl. Mater.* **454**, 381–386.

[bb5] Bacon, G. E. (1975). *Neutron Diffraction.* Oxford: Clarendon Press.

[bb6] Blau, W., Mager, S. & Wieser, E. (1977). *Phys. Status Solidi B* **81**, 535–544.

[bb7] Briggs, S. A., Edmondson, P. D., Littrell, K. C., Yamamoto, Y., Howard, R. H., Daily, C. R., Terrani, K. A., Sridharan, K. & Field, K. G. (2017). *Acta Mater.* **129**, 217–228.

[bb8] Certain, A. G., Field, K. G., Allen, T. R., Miller, M. K., Bentley, J. & Busby, J. T. (2010). *J. Nucl. Mater.* **407**, 2–9.

[bb9] Chang, K., Meng, F., Ge, F., Zhao, G., Du, S. & Huang, F. (2019). *J. Nucl. Mater.* **516**, 63–72.

[bb10] Cunningham, N. J., Wu, Y., Etienne, A., Haney, E. M., Odette, G. R., Stergar, E., Hoelzer, D. T., Kim, Y. D., Wirth, B. D. & Maloy, S. A. (2014). *J. Nucl. Mater.* **444**, 35–38.

[bb11] Dryepondt, S., Unocic, K. A., Hoelzer, D. T., Massey, C. P. & Pint, B. A. (2018). *J. Nucl. Mater.* **501**, 59–71.

[bb12] Dryepondt, S., Unocic, K. A., Hoelzer, D. T. & Pint, B. A. (2014). *Advanced ODS FeCrAl alloys for accident-tolerant fuel cladding.* Technical Manual ORNL/TM-2014/380. Oak Ridge National Laboratory, Oak Ridge, Tennessee, USA.

[bb13] Edmondson, P. D., Briggs, S. A., Yamamoto, Y., Howard, R. H., Sridharan, K., Terrani, K. A. & Field, K. G. (2016). *Scr. Mater.* **116**, 112–116.

[bb14] Fallot, M. (1936). *Ann. Phys.* **11**, 305–387.

[bb15] Field, K. G., Hu, X., Littrell, K. C., Yamamoto, Y. & Snead, L. (2015). *J. Nucl. Mater.* **465**, 746–755.

[bb16] Field, K. G., Littrell, K. C. & Briggs, S. A. (2018). *Scr. Mater.* **142**, 41–45.

[bb17] Field, K. G., McDuffee, J. L., Geringer, J. W., Petrie, C. M. & Katoh, Y. (2019). *Nucl. Instrum. Methods Phys. Res. B* **445**, 46–56.

[bb18] Gault, B., Moody, M. P., Cairney, J. M. & Ringer, S. P. (2012). *Atom probe microscopy.* Heidelberg: Springer.

[bb19] Glatter, O. (1977). *J. Appl. Cryst.* **10**, 415–421.

[bb20] Glatter, O. (1980). *J. Appl. Cryst.* **13**, 7–11.

[bb21] Gray, D. (1990). *Usage summary for selected optimization routines.* Computing Science Technical Report 153. AT&T Bell Laboratories, Murray Hill, New Jersey, USA.

[bb22] Gussev, M. N., Cakmak, E. & Field, K. G. (2018). *J. Nucl. Mater.* **504**, 221–233.

[bb23] Gussev, M. N., Field, K. G. & Yamamoto, Y. (2017*a*). *Mater. Des.* **129**, 227–238.

[bb24] Gussev, M. N., Howard, R. H., Terrani, K. A. & Field, K. G. (2017*b*). *Nucl. Eng. Des.* **320**, 298–308.

[bb25] Han, Y.-S., Mao, X., Jang, J. & Kim, T.-K. (2014). *Appl. Phys. A* **119**, 249–252.

[bb26] Hansen, S. & Pedersen, J. S. (1991). *J. Appl. Cryst.* **24**, 541–548.

[bb27] Hatzoglou, C., Radiguet, B., Da Costa, G., Pareige, P., Roussel, M., Hernandez-Mayoral, M. & Pareige, C. (2019). *J. Nucl. Mater.* **522**, 64–73.

[bb28] Hyde, J. M., Burke, M. G., Smith, G. D. W., Styman, P., Swan, H. & Wilford, K. (2014). *J. Nucl. Mater.* **449**, 308–314.

[bb29] Kinning, D. J. & Thomas, E. L. (1984). *Macromolecules* **17**, 1712–1718.

[bb30] Kobayashi, S. & Takasugi, T. (2010). *Scr. Mater.* **63**, 1104–1107.

[bb31] Mao, K. S., Massey, C. P., Yamamoto, Y., Unocic, K. A., Gussev, M. N., Zhang, D., Briggs, S. A., Karakoc, O., Nelson, A. T., Field, K. G. & Edmondson, P. D. (2022). *Acta Mater.* **231**, 117843.

[bb32] Massey, C. P., Dryepondt, S. N., Edmondson, P. D., Frith, M. G., Littrell, K. C., Kini, A., Gault, B., Terrani, K. A. & Zinkle, S. J. (2019*a*). *Acta Mater.* **166**, 1–17.

[bb33] Massey, C. P., Edmondson, P. D., Field, K. G., Hoelzer, D. T., Dryepondt, S. N., Terrani, K. A. & Zinkle, S. J. (2019*b*). *Scr. Mater.* **162**, 94–98.

[bb34] Massey, C. P., Zhang, D., Briggs, S. A., Edmondson, P. D., Yamamoto, Y., Gussev, M. N. & Field, K. G. (2021). *J. Nucl. Mater.* **549**, 152804.

[bb35] Mathon, M. H., de Carlan, Y., Geoffroy, G., Averty, X., Alamo, A. & de Novion, C. H. (2003). *J. Nucl. Mater.* **312**, 236–248.

[bb36] Mathon, M. H., Perrut, M., Zhong, S. Y. & de Carlan, Y. (2012). *J. Nucl. Mater.* **428**, 147–153.

[bb37] McMurray, J. W., Hu, R., Ushakov, S. V., Shin, D., Pint, B. A., Terrani, K. A. & Navrotsky, A. (2017). *J. Nucl. Mater.* **492**, 128–133.

[bb38] Ohnuma, M., Suzuki, J., Ohtsuka, S., Kim, S.-W., Kaito, T., Inoue, M. & Kitazawa, H. (2009). *Acta Mater.* **57**, 5571–5581.

[bb39] Pedersen, J. S. (1994). *J. Appl. Cryst.* **27**, 595–608.

[bb40] Pedersen, J. S. (1997). *Adv. Colloid Interface Sci.* **70**, 171–210.

[bb41] Prakash Kolli, R. & Seidman, D. N. (2007). *Microsc. Microanal.* **13**, 272–284. 10.1017/S1431927607070675 17637076

[bb42] Preston, G. D. (1932). *London Edinb. Dubl. Philos. Mag. J. Sci.* **13**, 419–425.

[bb43] Redlich, O. & Kister, A. T. (1944). *Trans. Am. Inst. Chem. Eng.* **44**, 345–348.

[bb44] Reese, E. R., Bachhav, M., Wells, P., Yamamoto, T., Robert Odette, G. & Marquis, E. A. (2018). *J. Nucl. Mater.* **500**, 192–198.

[bb45] Ribis, J. & Lozano-Perez, S. (2012). *Mater. Lett.* **74**, 143–146.

[bb46] Sequeira, A. D., Calderon, H. A., Kostorz, G. & Pedersen, J. S. (1995). *Acta Metall. Mater.* **43**, 3427–3439.

[bb47] Simm, T., Sun, L., Galvin, D., Hill, P., Rawson, M., Birosca, S., Gilbert, E., Bhadeshia, H. & Perkins, K. (2017). *Materials* **10**, 1346. 10.3390/ma10121346 PMC5744281 29168800

[bb48] Svergun, D. I. & Pedersen, J. S. (1994). *J. Appl. Cryst.* **27**, 241–248.

[bb49] Taylor, A. & Jones, I. M. (1958). *J. Phys. Chem. Solids* **6**, 16–37.

[bb50] Tsao, C.-S., Lin, T.-L. & Yu, M.-S. (1999). *J. Alloys Compd.* **289**, 81–87.

[bb51] Wharry, J., Swenson, M. & Yano, K. (2017). *J. Nucl. Mater.* **486**, 11–20.

[bb52] Wignall, G. D., Littrell, K. C., Heller, W. T., Melnichenko, Y. B., Bailey, K. M., Lynn, G. W., Myles, D. A., Urban, V. S., Buchanan, M. V., Selby, D. L. & Butler, P. D. (2012). *J. Appl. Cryst.* **45**, 990–998.

[bb53] Yamamoto, Y., Pint, B. A., Terrani, K. A., Field, K. G., Yang, Y. & Snead, L. L. (2015). *J. Nucl. Mater.* **467**, 703–716.

[bb54] Yamane, H., Omori, M. & Hirai, T. (1995). *J. Mater. Sci. Lett.* **14**, 470–473.

[bb55] Zhang, D., Briggs, S. A., Edmondson, P. D., Gussev, M. N., Howard, R. H. & Field, K. G. (2019). *J. Nucl. Mater.* **527**, 151784.

