# Supplementary material for: Shielded magnetic small-angle neutron scattering for characterization of radioactive samples
Source: J Appl Crystallogr. 2025 May 31;58(Pt 3):1000–14. doi: 10.1107/S1600576725003176 (PMC12135976; doi:10.1107/S1600576725003176)
Supplement: Supplementary file 1 [file j-58-01000-sup1.pdf]

JOURNAL OF APPLIED CRYSTALLOGRAPHY

**Volume 58 (2025)**

**Supporting information for article:**

# Shielded magnetic small-angle neutron scattering for characterization of radioactive samples

**Kevin G. Field, Caleb P. Massey, Kurt R. Smith, Samuel A. Briggs, Dalong Zhang and Kenneth C. Littrell**

**Table S1**: Lattice parameters of pure α-Fe, Cr and Al, as well as the fitted interaction parameters used in the calculation of the lattice parameter of an FeCrAl matrix based on the FeCrAl alloy's Cr and Al contents.

| Interaction parameter | Value (Å) | Reference |
|---|---|---|
| $a_{0,\alpha-Fe}$ | 2.866 | Abrahamson & Lopata (1966) |
| $a_{0,Cr}$ | 2.878 | Preston (1932) |
| $a_{0,Al}$ | 3.250 | Chang *et al.* (2019) |
| $L^0_{Fe,Cr}$ | 0.017 | This study |
| $L^1_{Fe,Cr}$ | 0.064 | This study |
| $L^0_{Fe,Al}$ | -0.586 | This study |
| $L^1_{Fe,Al}$ | -0.470 | This study |
| $L_{Fe,Cr,Al}$ | -0.619 | This study |

**Table S2:** Cluster characteristics for 125YF and C35M3 before and after irradiation as determined using either a simplistic approach or using a set of cubic spline basis functions, where *n* is the number of splines used to fit the APT-determined size distributions. Values in parentheses are the standard deviations of the observations for the simplistic approach and the Monte Carlo–based error analysis for the cubic spline fitting.

| Alloy | Cond. | Cluster Type | Radius Type | Simplified | | | $n=n_{SANS}$ | | | $n=n_{SANS}\times2$ | | |
|---|---|---|---|---|---|---|---|---|---|---|---|---|
| | | | | $R_p$ (nm) | $N_p \times 10^{23}$ ($m^{-3}$) | $\eta$ | $R_p$ (nm) | $N_p \times 10^{23}$ ($m^{-3}$) | $\eta$ | $R_p$ (nm) | $N_p \times 10^{23}$ ($m^{-3}$) | $\eta$ |
| 125YF | AsR | | $R_g$ | 1.5 (0.5) | | 0.009 (0.003) | 1.5 (0.0) | 7.0 (0.8) | 0.015 (0.003) | 1.5 (0.1) | 6.7 (0.9) | 0.014 (0.003) |
| | | Y,Al,O | $R_s$ | 2.0 (0.6) | 5.9 (1.0) | 0.019 (0.007) | 1.9 (0.1) | 6.9 (0.9) | 0.034 (0.006) | 2.0 (0.1) | 6.3 (0.7) | 0.029 (0.006) |
| | | | $R_a$ | 1.6 (0.6) | | 0.011 (0.005) | 1.6 (0.1) | 6.3 (0.9) | 0.018 (0.003) | 1.6 (0.1) | 6.3 (0.9) | 0.018 (0.003) |
| | Irr. | | $R_g$ | 1.6 (0.6) | | 0.010 (0.004) | 1.5 (0.0) | 6.1 (0.4) | 0.015 (0.002) | 1.6 (0.0) | 5.7 (0.3) | 0.015 (0.001) |
| | | Y,Al,O | $R_s$ | 2.1 (0.8) | 5.3 (0.2) | 0.021 (0.008) | 2.0 (0.0) | 6.1 (0.3) | 0.035 (0.003) | 2.1 (0.0) | 5.4 (0.3) | 0.032 (0.003) |
| | | | $R_a$ | 1.8 (0.7) | | 0.014 (0.005) | 1.8 (0.0) | 5.6 (0.3) | 0.015 (0.002) | 1.8 (0.0) | 5.4 (0.3) | 0.021 (0.002) |
| | | | $R_g$ | 1.4 (0.3) | | 0.001 (0.000) | 1.4 (0.0) | 0.7 (0.1) | 0.001 (0.000) | 1.4 (0.1) | 0.6 (0.1) | 0.001 (0.000) |
| | | Cr-rich | $R_s$ | 1.8 (0.4) | 0.6 (0.2) | 0.001 (0.001) | 1.8 (0.1) | 0.6 (0.1) | 0.002 (0.000) | 1.8 (0.1) | 0.6 (0.1) | 0.002 (0.000) |
| | | | $R_a$ | 1.8 (0.4) | | 0.001 (0.001) | 1.7 (0.1) | 0.7 (0.1) | 0.002 (0.000) | 1.8 (0.1) | 0.6 (0.1) | 0.002 (0.000) |
| | | | $R_g$ | 1.6 (0.6) | | 0.010 (0.004) | 1.5 (0.0) | 6.8 (0.4) | 0.016 (0.002) | 1.5 (0.0) | 6.3 (0.3) | 0.016 (0.001) |
| | | Y,Al,O + Cr-rich | $R_s$ | 2.1 (0.7) | 5.9 (0.2) | 0.022 (0.008) | 2.0 (0.0) | 6.7 (0.3) | 0.037 (0.003) | 2.1 (0.0) | 6.0 (0.3) | 0.034 (0.003) |
| | | | $R_a$ | 1.8 (0.7) | | 0.015 (0.006) | 1.8 (0.0) | 6.2 (0.3) | 0.024 (0.003) | 1.8 (0.0) | 6.0 (0.3) | 0.025 (0.002) |
| C35M | Irr. | | $R_g$ | 1.6 (0.6) | | 0.052 (0.025) | 1.5 (0.0) | 31.1 (1.9) | 0.066 (0.004) | 1.5 (0.0) | 30.2 (1.8) | 0.065 (0.004) |
| | | Cr-rich | $R_s$ | 2.1 (0.7) | 29.5 (9.8) | 0.112 (0.055) | 1.9 (0.0) | 30.3 (1.6) | 0.141 (0.008) | 2.0 (0.0) | 30.0 (1.5) | 0.141 (0.007) |
| | | | $R_a$ | 1.5 (0.6) | | 0.046 (0.023) | 1.4 (0.0) | 30.8 (2.3) | 0.057 (0.004) | 1.4 (0.0) | 30.8 (2.4) | 0.056 (0.003) |

*Notation*: AsR: As-received; Irr.: Irradiated, $R_g$: Radius of gyration, $R_s$: Radius of spherical equivalent; $R_a$: Radius of atomic count; $R_p$: mean radius of precipitate/cluster; $N_p$: Number density; η: Volume fraction; nSANS: number of splines used in SANS analysis

**Table S3**: Calculated hard sphere interaction values and volume fractions using the FF fitting technique on the magnetic shielded SANS data.

| Alloy | Condition | $C_{hs}$ | $\eta_{hs}$ | $\eta = \eta_{hs}(C_{hs})^{-3}$ | $\eta = \frac{4\pi \overline{r^3}}{3}$ |
|---|---|---|---|---|---|
| 125YF | As-received | 1.46 (0.10) | 0.58 (0.08) | 0.188 (0.060) | 0.014 (0.004) |
| | Irradiated | 2.53 (0.14) | 0.53 (0.12) | 0.032 (0.015) | 0.011 (0.001) |
| C35M | As-received | N.M. | N.M. | N.M. | N.M. |
| | Irradiated | 1.19 (0.00) | 0.53 (0.00) | 0.311 (0.000) | 0.107 (0.002) |

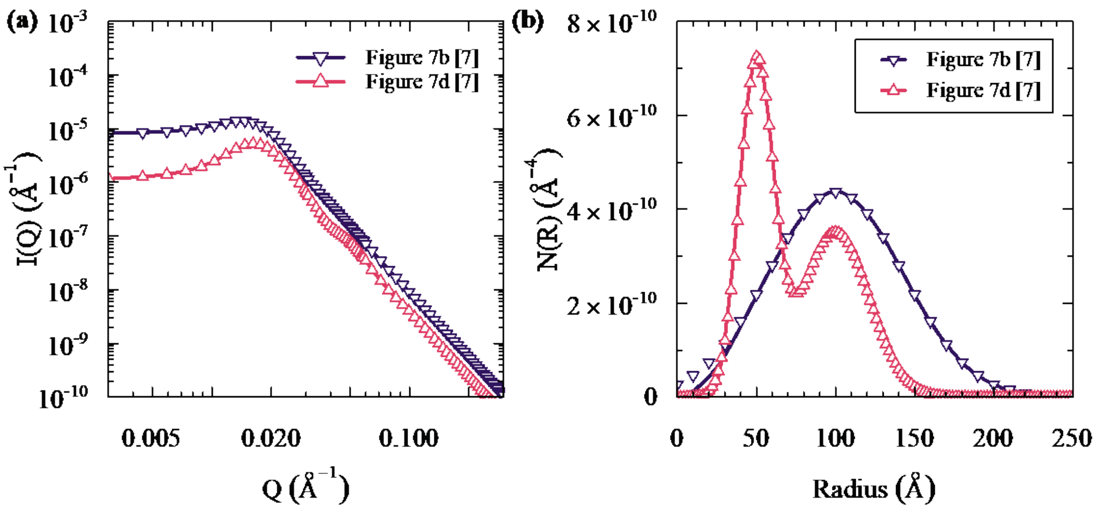


**Figure S1**: Comparison between the simulated small-angle scattering data and size distributions (symbols) from selected examples within Pedersen's local monodispersed approximation (Pedersen, 1994) and the data fitted using the computer routine (lines) described in Section 2.4 showing good recovery of the data, except in the size distributions below 30 Å due to numerical instabilities in the non-negative least-squares solver. Error is below the line width in the size distributions when determined using the Monte Carlo method. Values for C, $\eta_{hs}$, and $\bar{r}$ through the fitting routine were found to be <5% to the simulated values.

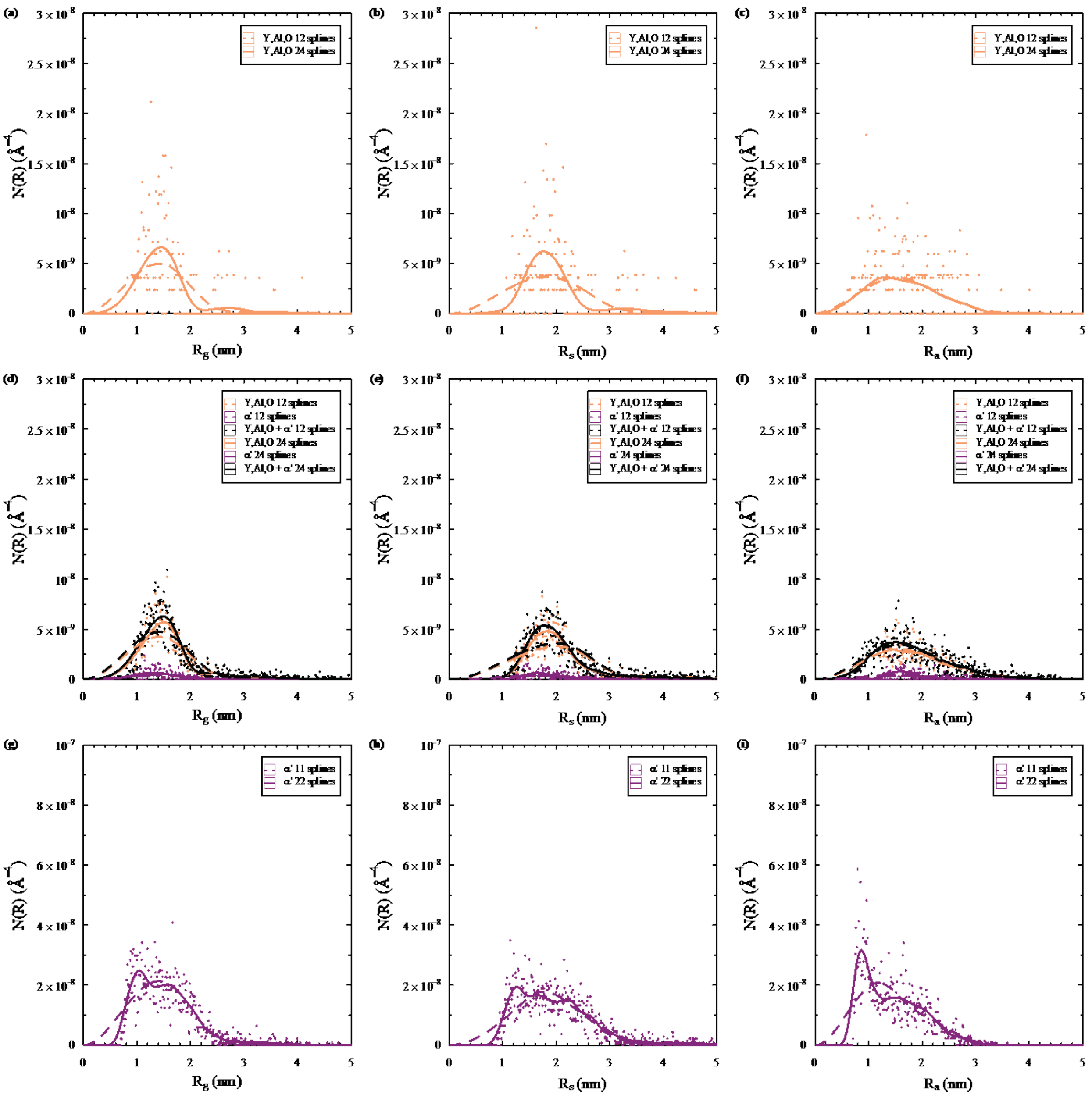


**Figure S2**: Comparison between (a, d, g) radius of gyration $R_g$, (b, e, h) radius of spherical equivalent $R_s$, and (c, f, i) radius derived from the atomic count $R_a$ size distributions extracted from the APT data (points) and the corresponding size distributions calculated by fitting the same size distribution functions using equation (13) (lines), where *N* is equal to the number of splines given in the legend for (a–c) 125YF specimen in the as-received state, (d–f) 125YF specimen after irradiation to 1.8 dpa at 357°C and (g–i) C35M specimen after irradiation to 1.8 dpa at 357°C. Shaded areas in size distributions represent the 95% confidence interval from Monte Carlo–based error analysis.
